# High‐throughput automated scoring of Ki67 in breast cancer tissue microarrays from the Breast Cancer Association Consortium

**DOI:** 10.1002/cjp2.42

**Published:** 2016-04-06

**Authors:** Mustapha Abubakar, William J Howat, Frances Daley, Lila Zabaglo, Leigh‐Anne McDuffus, Fiona Blows, Penny Coulson, H Raza Ali, Javier Benitez, Roger Milne, Herman Brenner, Christa Stegmaier, Arto Mannermaa, Jenny Chang‐Claude, Anja Rudolph, Peter Sinn, Fergus J Couch, Rob A.E.M. Tollenaar, Peter Devilee, Jonine Figueroa, Mark E Sherman, Jolanta Lissowska, Stephen Hewitt, Diana Eccles, Maartje J Hooning, Antoinette Hollestelle, John WM Martens, Carolien HM van Deurzen, kConFab Investigators, Manjeet K Bolla, Qin Wang, Michael Jones, Minouk Schoemaker, Annegien Broeks, Flora E van Leeuwen, Laura Van't Veer, Anthony J Swerdlow, Nick Orr, Mitch Dowsett, Douglas Easton, Marjanka K Schmidt, Paul D Pharoah, Montserrat Garcia‐Closas

**Affiliations:** ^1^ Division of Genetics and EpidemiologyThe Institute of Cancer ResearchLondonUK; ^2^ Cancer Research UK Cambridge Institute, University of CambridgeCambridgeUK; ^3^ Breakthrough Breast Cancer Research Centre, Division of Breast Cancer Research, The Institute of Cancer ResearchLondonUK; ^4^ Academic Department of Biochemistry, Royal Marsden HospitalFulham RoadLondon; ^5^ Centre for Cancer Genetic Epidemiology, Department of Oncology, University of CambridgeCambridgeUK; ^6^ Human Genetics Group, Human Cancer Genetics Program, Spanish National Cancer Research Centre (CNIO)MadridSpain; ^7^ Centro de Investigacion en Red de Enfermedades Raras (CIBERER)ValenciaSpain; ^8^ Cancer Epidemiology Centre, Cancer Council VictoriaMelbourneAustralia; ^9^ Centre for Epidemiology and Biostatistics, Melbourne School of Population and Global health, The University of MelbourneMelbourneAustralia; ^10^ Division of Clinical Epidemiology and Aging Research, German Cancer Research Center (DKFZ)HeidelbergGermany; ^11^ Division of Preventive Oncology, German Cancer Research Center (DKFZ), and National Center for Tumor Diseases (NCT)HeidelbergGermany; ^12^ German Cancer Consortium (DKTK), German Cancer Research Center (DKFZ)HeidelbergGermany; ^13^ Saarland Cancer RegistrySaarlandGermany; ^14^ School of Medicine, Institute of Clinical Medicine, Pathology and Forensic Medicine, Cancer Center of Eastern Finland, University of Eastern FinlandKuopioFinland; ^15^ Imaging Center, Department of Clinical Pathology, Kuopio University HospitalKuopioFinland; ^16^ Division of Cancer Epidemiology, German Cancer Research Center (DKFZ)HeidelbergGermany; ^17^ University Cancer Center Hamburg (UCCH), University Medical Center Hamburg‐EppendorfHamburgGermany; ^18^ Department of PathologyInstitute of Pathology, Heidelberg University HospitalGermany; ^19^ Department of Laboratory Medicine and PathologyMayo ClinicRochester, MNUSA; ^20^ Department of SurgeryLeiden University Medical CenterThe Netherlands; ^21^ Department of Human Genetics & Department of PathologyLeiden University Medical CenterLeidenThe Netherlands; ^22^ Usher Institute of Population Health Sciences and Informatics, The University of EdinburghScotlandUK; ^23^ Division of Cancer Epidemiology and GeneticsNational Cancer InstituteRockvilleMarylandUSA; ^24^ Department of Cancer Epidemiology and PreventionM. Sklodowska‐Curie Memorial Cancer Center and Institute of OncologyWarsawPoland; ^25^ Laboratory of PathologyNational Cancer Institute, National Institutes of HealthRockvilleMDUSA; ^26^ Faculty of Medicine Academic Unit of Cancer SciencesSouthampton General HospitalSouthamptonUK; ^27^ Family Cancer Clinic, Department of Medical Oncology, Erasmus MC Cancer InstituteRotterdamThe Netherlands; ^28^ Department of PathologyErasmus MC Cancer InstituteRotterdamThe Netherlands; ^29^ Department of GeneticsQIMR Berghofer Medical Research InstituteBrisbaneAustralia; ^30^ Centre for Cancer Genetic Epidemiology, Department of Public Health and Primary Care, University of CambridgeCambridgeUK; ^31^ Division of Molecular PathologyNetherlands Cancer Institute, Antoni van Leeuwenhoek HospitalAmsterdamThe Netherlands; ^32^ Division of Psychosocial Research and EpidemiologyNetherlands Cancer Institute, Antoni van Leeuwenhoek HospitalAmsterdamThe Netherlands; ^33^ Division of Breast Cancer ResearchThe Institute of Cancer ResearchLondonUK

**Keywords:** breast cancer, automated algorithm, tissue microarrays, Ki67, immunohistochemistry

## Abstract

Automated methods are needed to facilitate high‐throughput and reproducible scoring of Ki67 and other markers in breast cancer tissue microarrays (TMAs) in large‐scale studies. To address this need, we developed an automated protocol for Ki67 scoring and evaluated its performance in studies from the Breast Cancer Association Consortium. We utilized 166 TMAs containing 16,953 tumour cores representing 9,059 breast cancer cases, from 13 studies, with information on other clinical and pathological characteristics. TMAs were stained for Ki67 using standard immunohistochemical procedures, and scanned and digitized using the Ariol system. An automated algorithm was developed for the scoring of Ki67, and scores were compared to computer assisted visual (CAV) scores in a subset of 15 TMAs in a training set. We also assessed the correlation between automated Ki67 scores and other clinical and pathological characteristics. Overall, we observed good discriminatory accuracy (AUC = 85%) and good agreement (kappa = 0.64) between the automated and CAV scoring methods in the training set. The performance of the automated method varied by TMA (kappa range= 0.37–0.87) and study (kappa range = 0.39–0.69). The automated method performed better in satisfactory cores (kappa = 0.68) than suboptimal (kappa = 0.51) cores (*p*‐value for comparison = 0.005); and among cores with higher total nuclei counted by the machine (4,000–4,500 cells: kappa = 0.78) than those with lower counts (50–500 cells: kappa = 0.41; *p*‐value = 0.010). Among the 9,059 cases in this study, the correlations between automated Ki67 and clinical and pathological characteristics were found to be in the expected directions. Our findings indicate that automated scoring of Ki67 can be an efficient method to obtain good quality data across large numbers of TMAs from multicentre studies. However, robust algorithm development and rigorous pre‐ and post‐analytical quality control procedures are necessary in order to ensure satisfactory performance.

## Introduction

Breast cancer is not a single entity but a heterogeneous disease 1, 2, characterized by subtypes which differ not only in terms of outcome 3, 4 but also aetiologically 5, 6. Over the years, epidemiologists have sought to investigate aetiological and/or prognostic heterogeneity among immunohistochemically defined subtypes of the disease. Recently, along with other immunohistochemical (IHC) markers, Ki67 has been recommended for use in the surrogate definition of the intrinsic subtypes of breast cancer 7, 8. Incorporating Ki67 and other IHC markers into large, multicentre, epidemiological studies into breast cancer subtypes requires high‐throughput standardized scoring of tissue markers.

Visual and automated approaches have been suggested as ways to address the challenge of large‐scale scoring of IHC markers in breast cancer 9. Visual scoring can be achieved on a large scale by the utilization of multiple scorers or via web‐based platforms that allow scoring to be performed by several expert scorers from different locations. Recently, the potential usefulness of crowdsourcing of the general public for the scoring of tissue markers has equally been evaluated 10. While visual scoring may ensure accuracy in recognition of tumour cells versus benign ductal epithelial or stromal cells and in the implementation of quality control protocols, it is often difficult to organize, slow, laborious and, for almost all of the markers, exhibits varying degrees of intra‐ and inter‐observer reproducibility. This is even more so for Ki67 for which a number of studies have reported poor inter‐observer reproducibility 11, 12, 13. On the other hand, automated algorithms are high‐throughput and reproducible, and several investigators have reported evidence in support of their use for the scoring of tissue markers especially oestrogen receptor (ER), progesterone receptor (PR), human epidermal growth factor receptor 2 (HER2) 14, 15, 16, 17, 18, 19, B‐cell CLL/lymphoma 2 (BCL2) 17, 20, epidermal growth factor receptor (EGFR) 18, 21, 22, cytokeratin (CK) 5/6 18 and Ki67 13, 23, 24, 25, 26, 27, 28.

However, unlike ER, PR and HER2, few studies have investigated the performance of automated scoring algorithms for the unsupervised scoring of Ki67 in tissue microarrays (TMAs) from large consortia. This is necessary given the heterogeneity in pre‐analytical variables (including TMA designs, tissue fixation, TMA age, and staining protocols) that is inherent in such study designs. Furthermore, It has now been shown that the performance of automated methods can vary by TMA 18 and potentially also according to other pre‐analytical variables 29. To our knowledge, most of the studies that have previously investigated the usefulness of automated scoring for Ki67 were single centre studies, thus were unable to assess the utility of such methods in the large‐scale scoring of Ki67 in TMAs from diverse populations. In this study, we developed and applied an automated protocol for the scoring of Ki67 in TMAs from multiple study centres within the Breast Cancer Association Consortium (BCAC). Using the resulting data, we assessed the associations between automated Ki67 scores and other clinical and pathological characteristics and how these compare with what has been reported in the literature.

## Materials and methods

### Study populations and study design

**Figure 1:**
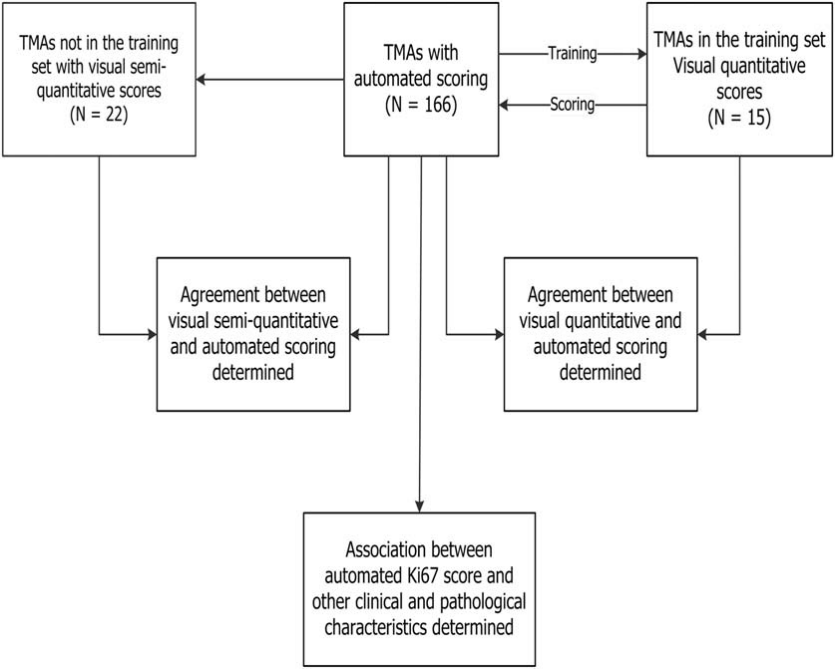
Study design. Of the 166 TMAs, 15 were selected as the training set and were used to develop an algorithm that was applied to the scoring of all 166 TMAs, containing 16,953 tissue cores. The agreements between automated and visual scores were determined for the TMAs in the training set. Furthermore, a subset of the TMAs (*N* = 22) had pathologists' semi quantitative Ki67 scores: as a result, automated scores from these were compared with the pathologists' scores and the agreement between the two also determined. In the next stage of the study, scores derived using the automated method were combined with information on other clinical and pathological characteristic for all subjects in the study (*N* = 9,059). The distribution of Ki67 scores across categories and its association with pathological characteristics were then determined.

BCAC is a large ongoing collaborative project of breast cancer studies involving study groups across the world 30. For the current study, we collected 166 TMAs from 13 participating studies based on the availability of tumour material on TMAs (Table 1). Ten studies (ABCS, CNIO, ESTHER, KBCP, MCBCS, ORIGO, POSH, RBCS, UKBGS and kConFab) submitted unstained TMA slides which were centrally stained in the Breakthrough Core Pathology Laboratory at the Institute of Cancer Research (ICR) while two studies (MARIE and PBCS) submitted TMAs stained at their local laboratories. One study (SEARCH) submitted Ariol digital images acquired using a similar technology to the one at the ICR. Digitization and centralized automated scoring of all the TMAs was performed at the ICR. All study groups provided data on other clinical and pathological characteristics for each patient. These data were centrally queried and quality checked at the NKI‐AVL in Amsterdam. In addition, the PBCS study provided semi‐quantitative visual scores while the SEARCH study provided categories of visual scores corresponding to Allred proportions. In terms of study design, Figure 1 shows the 166 TMAs, 15 of which containing 1,346 cores were selected as the training set. These were used to develop an algorithm that was then applied to the scoring of all 166 TMAs and the resulting automated scores analysed to determine agreement with pathologists’ scores and association with other clinico‐pathological variables.

**Table 1:** Description of the source populations, numbers of cases and designs of TMAs used in this study

Study acronym	Country	Cases (*N*)	Age at diagnosis mean (range)	TMAs	Cores per case	Cores per TMA	Core size (mm)	Total cores per study
ABCS	Netherlands	892	43 (19–50)	24	1–6	15–328	0.6	2,449
CNIO	Spain	164	60 (35–81)	4	1–2	80–133	1.0	316
ESTHER	Germany	258	62 (50–75)	6	1–2	78–91	0.6	461
KBCP	Finland	276	59 (30–92)	12	1–3	63–94	1.0	724
MARIE	Germany	808	62 (50–75)	27	1–5	32–92	0.6	1,490
MCBCS	USA	491	58 (22–87)	7	1–8	131–301	0.6	1,630
ORIGO	Netherlands	383	53 (22–87)	9	1–9	67–223	0.6	991
PBCS	Poland	1,236	56 (27–75)	22	1–2	66–145	1.0	2,358
POSH	UK	73	36 (27–41)	5	1–5	75–114	0.6	194
RBCS	Netherlands	234	45 (25–84)	6	1–5	134–199	0.6	642
SEARCH	UK	3,528	52 (24–70)	24	1–3	120–167	0.6	4,037
UKBGS	UK	367	56 (24–84)	14	1–4	62–114	1.0	1,130
kConFab	Australia	349	45 (20–77)	6	1–2	65–114	0.6	531
Totals		9,059	56 (19–92)	166	1–9	15–328	0.6–1.0	16,953

### Ki67 immunostaining

Sections were dewaxed using xylene and rehydrated through graded alcohol (100, 90 and 70%) to water. Slides were then placed in a preheated (5 min 800 W microwave) solution of Dako Target Retrieval solution pH 6.0 (S1699) and microwaved on high power for 10 min and then allowed to cool in this solution at room temperature for 10 min. In the next stage, the slides were placed on a Dako Autostainer and stained using a standard protocol using Dako MIB‐1 diluted 1/50 and visualized using the Dako REAL kit (K5001). The MIB‐1 antibody was also adopted for the staining of those TMAs that were not part of those centrally stained at the ICR but at varying concentrations (PBCS = 1:500; MARIE = 1:400 and SEARCH = 1:200) (supplementary material, Table S1).

### Development of scoring protocol

#### Computer assisted visual scoring protocol

**Figure 2:**
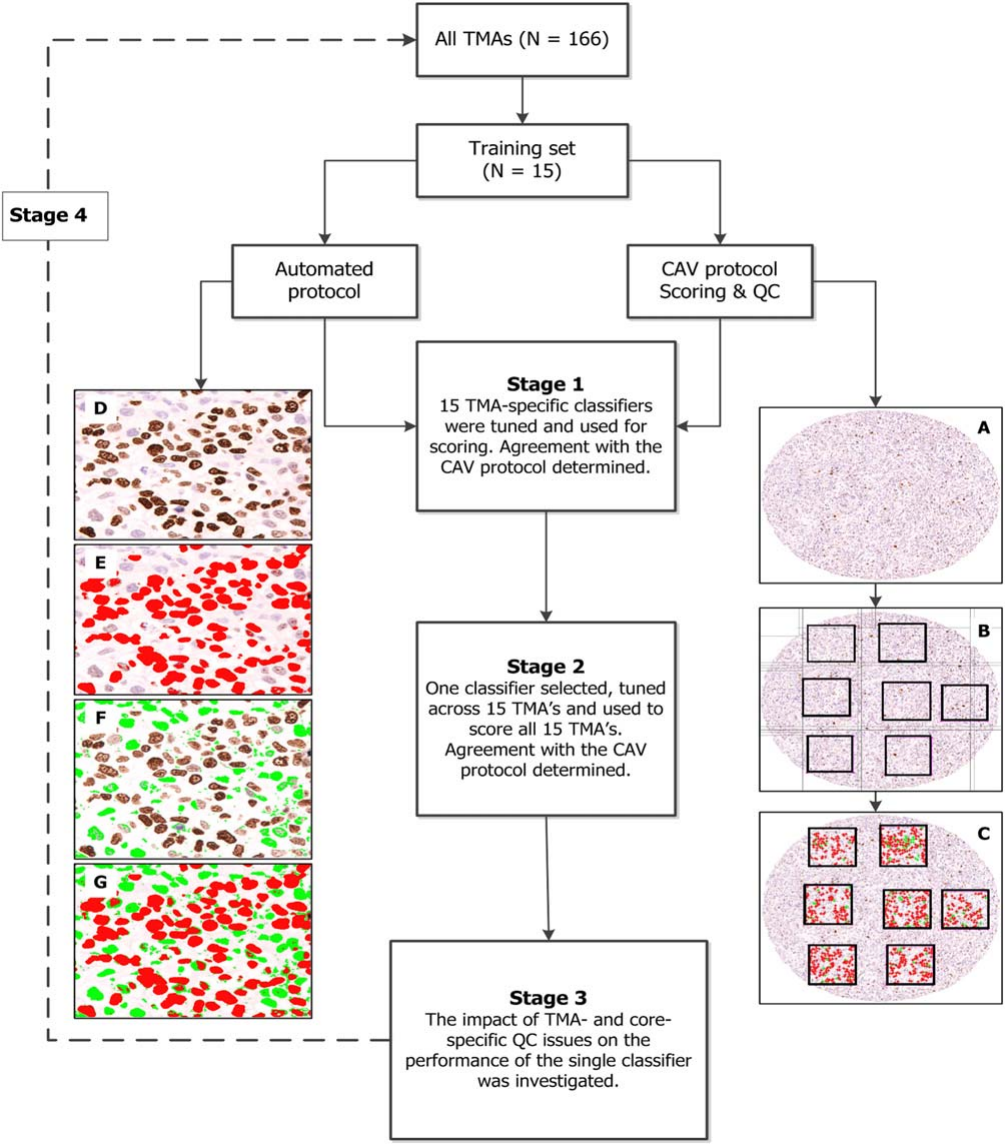
Schematic representation of the stages involved in the development of a centralised scoring protocol. Of the 166 TMAs, 15 were randomly selected as the training set. Two protocols were developed and adopted for scoring: A computer‐assisted visual (CAV) and automated scoring protocols. Using the CAV protocol, a grid was used to demarcate each core and at least six well‐delineated areas of the core were counted for positive and negative nuclei (right hand panel (A) tumour core; (B) demarcation into regions by a grid and (C) counting of positive and negative nuclei within the squares) and the average score obtained. For the automated scoring protocol (Stage 1), 15 TMA‐specific classifiers were tuned (left hand panel (D) region of interest, (E) colour detection of DAB/positive nuclei, (F) colour detection of haematoxylin/negative nuclei and (G) combined detection of positive and negative nuclei) and used for scoring. In the next stage (Stage 2) one classifier was selected, tuned further, and used to score all 15 TMAs. Agreement with the CAV protocol was further tested and the impact of quality control on the performance of this classifier was then assessed (Stage 3). In the final stage (Stage 4), this classifier was applied to the scoring of all 166 TMAs in this study.

All TMAs were digitized using the Ariol 50s digital scanning machine. Our computer assisted visual (CAV) approach to visual scoring uses the Ariol interface and software tools for consistent and reproducible counting of positive and negative tumour nuclei. This yielded quantitative visual scores which enabled direct comparison with automated scores in a manner similar to that reported by Laurinavicious *et al* 31. Using this approach, a grid was placed on each tumour core (Figure 2A) thereby delineating it into distinct regions (Figure 2B). Within each of these regions, a 250 µm by 250 µm square (each corresponding to a high power field (×40) under the microscope) was placed and the number of positive and negative malignant nuclei in each square counted (Figure 2B and C). This method prevents the double counting of positive and/or negative nuclei. The Ki67 score for each core was calculated as the percentage of positive nuclei across the entire spectrum of the core, including hot spots. This is in keeping with the recommendations of the International Ki67 in Breast Cancer working group 32. Modifications were made to the standard protocol to account for skewed distribution of tumour tissue within the core or unevenly infiltrating clusters or nests of malignant cells. Counting was performed by a pathologist (MA) and the intra‐observer reproducibility of the protocol was confirmed by re‐scoring a random subset of cores (*N* = 111) 3 months after the first time they were scored (observed agreement = 96%; kappa = 0.90). The inter‐observer agreement was evaluated by comparing Ki67 values from a randomly selected subset of cores across four TMAs (*N* = 202) scored using this method with those previously scored by two other scorers (scorer 2 and scorer 3) and this was found to be good (supplementary material, Table S2). Here, we refer to scores derived using this approach as the ‘CAV score’.

The CAV protocol was also used to assign quality control categories to cores as follows: (1) Invasive satisfactory core (nuclei count >500); (2) DCIS satisfactory core (nuclei count >500); (3) Suboptimal cores, ie, few tumour cells (<500 malignant nuclei), staining issues (membrane, cytoplasmic and/or background staining), folded/marginally distorted core, suboptimal fixation. For the purpose of further analysis, categories 1 and 2 were considered as ‘satisfactory’ while category 3 was considered ‘suboptimal’.

Visual scoring in the external TMAs was performed by two independent scorers (scorers 2 and 3) by assigning semi‐quantitative Ki67 percentages to cores (ie, 0%, 25%, 50%, 75% and 100%). The Ki67 score for each patient was then taken as the average score from the two scorers across all cores for that patient.

#### The automated scoring protocol

The Ariol machine has functionality that enables the automatic detection of malignant and non‐malignant cells using shape and size characteristics. Using colour deconvolution, it can also distinguish between DAB positive and negative (haematoxylin‐stained) malignant cells. Achieving this however requires the development of classifiers. At first, one classifier was tuned for each of the 15 TMAs in the training set (known here as ‘TMA‐specific’ classifiers). Training involved tuning colour and shape parameters across several regions of interest. To determine the negative and positive populations of cells, a region of interest (Figure 2D) was demarcated and two colours were selected to indicate positive and negative nuclear populations (red for positive nuclei – Figure 2E; and green for negative nuclei – Figure 2F). The appropriate colour pixels were then selected to represent the full range of hue, saturation and intensity that was considered representative of the positive and negative nuclear classes. Subsequently, the best shape parameters that discriminated malignant and non‐malignant cells according to their spot width, width, roundness, compactness and axis ratio were then also selected. The spot width marks the location of the nuclei and separates them by size. Larger values select for larger cells while excluding smaller cells. The width is useful in sorting cells based on their size while the compactness and roundness are useful in sorting cells based on how circular they are. The axis‐ratio uses the centre of gravity of an object relative to its edges to separate elongated objects from rounder ones; larger values of this exclude elongated objects.

The TMA 9 classifier, having showed the best agreement parameters with the CAV, was then selected and tuned further across other TMA regions to generate a single (Universal) classifier (supplementary material, Tables S3 and S4). This was then applied to the scoring of all 15 TMAs and the agreement with CAV re‐evaluated. Subsequently, the impact of quality control – including total nuclei counted per core – on the performance of the Universal classifier was determined. In the final stage, the Universal classifier was applied to the scoring of all TMAs in this study (Figure 2).

Pre‐analytical QC protocols included the identification of three control cores (ie, strongly positive, negative and blank) per TMA while post analytical QC protocols included the exclusion of cores with total nuclei count <50 or >15,000 and/or Ki67 score of exactly 100%.

### Statistical methods

The area under the curve (AUC) of the receiver operating characteristics graph was used to evaluate the discriminatory accuracy of the quantitative automated scores to distinguish between positive and negative visual cores dichotomized using the most commonly reported visual cut‐off point of 10% positive cells 33. The linearly weighted kappa statistic 34 was used to measure the agreement between semi‐quantitative automated and visual scores categorized into quartiles as follows: Q1 = <25th percentile; Q2 = 25th–50th percentile; Q3 = >50th–75th percentile and Q4 = >75th percentile. Frequency tables were used to evaluate categories showing marked discrepancy, ie, cases in which either the machine or the visual scorer scores a core Q4 and the other scores it Q1 or vice versa, overall and for each TMA (supplementary material, Table S5). Agreement analyses were stratified by classifier type (TMA‐specific versus Universal), quality control category (satisfactory versus suboptimal) and by total nuclei counted by the machine (categorized at intervals of 500). The subject‐level Ki67 score was calculated as the average score across all cores for that subject. These were used: firstly, to determine the subject‐level agreement between automated and pathologists’ semi quantitative scores for a subset of patients that had pathologists’ scores from the study groups; secondly, to determine the distribution of Ki67 across categories of other clinical and pathological characteristics; and thirdly, to test the association between Ki67 and other clinical and pathological characteristics. Automated Ki67 was dichotomized at a cut‐off point of 10% and the associations between dichotomous categories of Ki67 and other pathological characteristics were determined in logistic regression models adjusted for age at diagnosis and study group. All analyses were conducted using STATA 13.1 software (StataCorp, College Station, TX, USA), were two‐sided and *p* values of <0.05 were considered as significant.

## Results

### TMAs design and clinico‐pathological characteristics of cases

A total of 166 TMAs containing 19,039 tumour cores representing 10,005 patients were collected from the 13 collaborating studies. Of these, 2,086 cores representing 946 cases failed QC (9.9% ductal and 8.7% lobular). As a result, a total of 16,953 tumour cores from 9,059 breast cancer patients were evaluated in this analysis (Table 1). The average age at diagnosis in these studies was 56 years (range 43–62 years). The designs of the TMAs differed among the 13 study groups according to a number of characteristics including core size (range = 0.6–1 mm); number of cores per case (range = 1–9); and number of cores per TMA (range = 15–328) (Table 1).

### Agreement between automated and CAV methods among the 15 TMAs in the training set (*N* = 1,346 cores)

**Figure 3:**
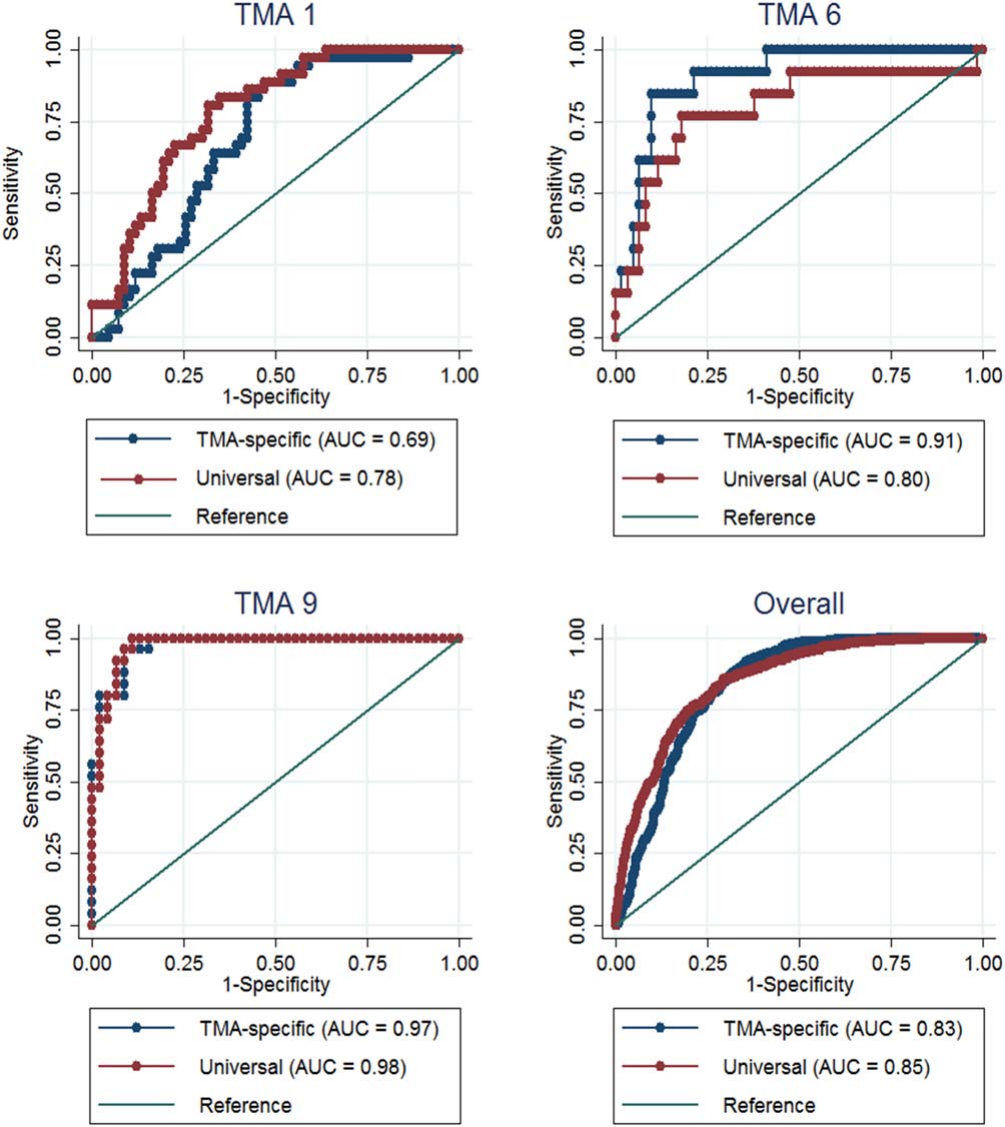
Graphs comparing the ROC curves for the discriminatory accuracy of the automated continuous Ki67 scores against categories of the visual score by classifier type (TMA‐specific and universal) among representative TMAs. In TMA 1, the universal classifier showed better discrimination than the TMA‐specific classifier; in TMA 6, the TMA‐specific classifier showed better discrimination while in TMA 9 no difference was observed between the two classifier types. Overall, both classifiers showed similar discriminatory accuracy.

**Figure 4:**
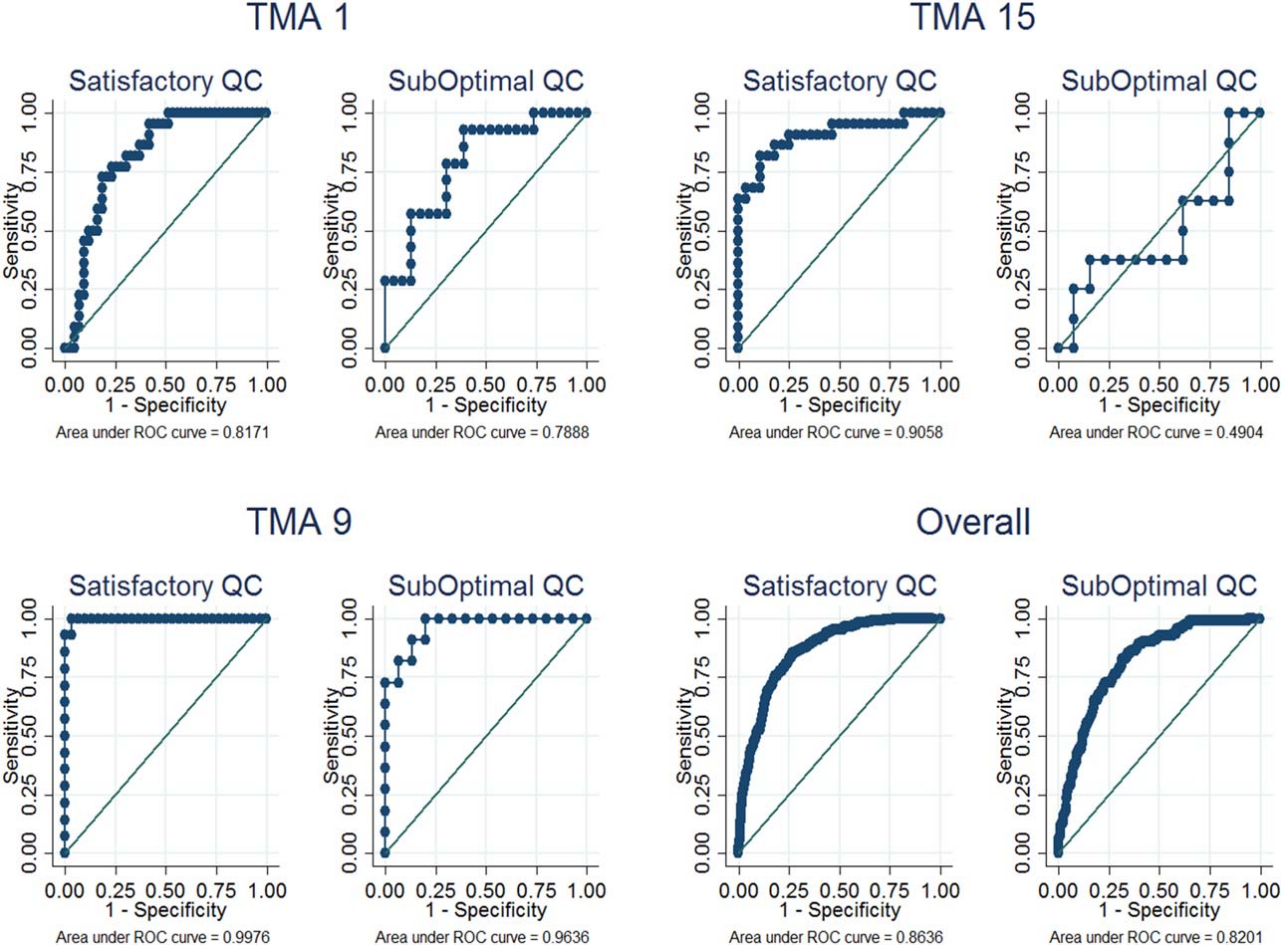
Graphs comparing the ROC curves for the discriminatory accuracy of the automated continuous scores against categories of the visual score by QC status among representative TMAs. The discriminatory accuracy was better among cores with satisfactory QC, overall and in TMAs 1 & 15. This difference was however not as obvious in TMA 9 as in 1 and 15.

The TMA‐specific classifier showed better accuracy than the Universal classifier in discriminating between visually determined positive and negative cores in eight of the 15 TMAs even though this was significant in only one of the TMAs (TMA 5, *p* = 0.04). On the other hand, the Universal classifier showed better kappa statistics in ten of the 15 TMAs (Table 2). Overall, good discriminatory accuracy (AUC (95% CI) = 83% (81–86%)) and moderate kappa agreement (agreement = 85%; kappa = 0.58) were observed between the TMA‐specific classifier and the CAV scores. This was slightly better for the Universal classifier which showed good discriminatory accuracy (AUC (95% CI) = 85% (83–87%)) and good agreement (agreement = 87%; kappa = 0.64) with the CAV scores. The overall performance of the TMA‐specific classifier was affected by three classifiers with low kappa values, ie, TMAs 1, 13 and 15. Heterogeneity was observed in the performance of the automated methods according to TMAs in both the TMA‐specific (range (AUC = 69–97%; agreement = 69–94%; kappa = 0.27–0.84)) and Universal (range (AUC = 78–98%; agreement = 80–96%; kappa = 0.37–0.87)) classifiers (Table 2, Figure 3 and supplementary material, Figure S1). Overall, the discriminatory accuracy and kappa agreement were better among satisfactory (AUC = 86%; agreement = 89%; kappa = 0.68) than suboptimal (AUC = 82%; agreement = 85%; kappa = 0.51) cores (*p* value for comparison = 0.005) and this pattern was seen in 11 of the 15 TMAs (Table 3, Figure 4 and supplementary material, Figure S2).

**Table 2:** Agreement parameters (observed agreement and kappa statistic) and discriminatory accuracy (AUC) parameters for visual and automated scores (derived using TMA‐specific and Universal classifiers) overall and for each of the 15 TMAs in the training set

TMA Name	*N*	TMA‐specific classifier			Universal classifier*		
		AUC (95% CI)	Observed agreement (95% CI)	Kappa (95% CI)	AUC (95% CI)	Observed agreement (95% CI)	Kappa (95% CI)
TMA 1	102	69 (59, 79)	73 (64, 82)	0.29 (0.21, 0.39)	78 (69, 87)	80 (71, 88)	0.37 (0.28, 0.47)
TMA 2	89	93 (88, 99)	82 (72, 89)	0.57 (0.45, 0.67)	91 (84, 97)	90 (82, 95)	0.75 (0.65, 0.84)
TMA 3	120	88 (82, 94)	87 (79, 92)	0.60 (0.51, 0.69)	86 (80, 93)	84 (75, 90)	0.49 (0.40, 0.58)
TMA 4	154	87 (81, 92)	91 (85, 95)	0.71 (0.64, 0.78)	83 (77, 90)	87 (81, 92)	0.58 (0.50, 0.66)
TMA 5	89	94 (88, 99)	93 (86, 97)	0.81 (0.71, 0.88)	87 (80, 95)	89 (82, 95)	0.69 (0.58, 0.78)
TMA 6	74	91 (83, 98)	89 (80, 95)	0.60 (0.47, 0.71)	80 (64, 96)	84 (73, 91)	0.44 (0.33, 0.57)
TMA 7	101	86 (79, 93)	89 (81, 94)	0.62 (0.52, 0.72)	88 (81, 95)	90 (83, 95)	0.67 (0.57, 0.76)
TMA 8	104	96 (93, 100)	84 (75, 90)	0.59 (0.49, 0.68)	91 (84, 97)	80 (71, 87)	0.37 (0.27, 0.47)
TMA 9	70	97 (95, 100)	94 (86, 98)	0.84 (0.74, 0.92)	98 (95, 100)	95 (86, 98)	0.85 (0.75, 0.93)
TMA 10	70	90 (83, 98)	93 (84, 98)	0.79 (0.67, 0.87)	94 (90, 99)	96 (88, 99)	0.87 (0.77, 0.94)
TMA 11	69	91 (84, 98)	90 (80, 96)	0.72 (0.60, 0.83)	89 (81, 97)	90 (80, 96)	0.73 (0.62, 0.84)
TMA 12	86	90 (83, 96)	85 (76, 92)	0.35 (0.25, 0.46)	91 (84, 97)	88 (80, 94)	0.47 (0.36, 0.58)
TMA 13	72	70 (58, 82)	69 (57, 80)	0.27 (0.17, 0.38)	84 (72, 96)	92 (83, 97)	0.73 (0.62, 0.83)
TMA 14	75	87 (79, 95)	75 (65, 85)	0.40 (0.29, 0.52)	85 (75, 94)	87 (77, 93)	0.64 (0.52, 0.75)
TMA 15	71	70 (57, 82)	82 (71, 90)	0.34 (0.23, 0.46)	80 (70, 91)	87 (77, 94)	0.56 (0.44, 0.68)
Overall	1,346	83 (81, 86)	85 (83, 87)	0.58 (0.55, 0.61)	85 (83, 87)	87 (86, 89)	0.64 (0.61, 0.66)

TMA‐specific classifiers represent automated algorithms that were trained specifically for each individual TMA. Universal classifier is a single automated algorithm tuned across the spectrum of TMAs in the training set and used for the scoring of all 15 TMAs. The Area Under the Curve (AUC) was determined by plotting a Receiver Operating Characteristic (ROC) curve of the continuous Ki67 automated score against categories of the visual scores – dichotomised using the most commonly reported cut‐off point in the literature of 10% (33)

The agreement and kappa statistics were determined by comparing quartiles (<25th, 25th–50th, >50th–75th and >75th percentiles) of both the visual and automated scores using weighted kappa statistics. *N*, Represents the number of cores on each TMA.

*The Universal classifier was adopted for use in the scoring of all TMAs (*N* = 166) in this study.

**Table 3:** Agreement (observed agreement, kappa statistic) and discriminatory accuracy (AUC) parameters for the automated and visual scores according to quality control status (satisfactory, *N* = 950 and suboptimal, *N* = 396) overall and among the 15 TMAs in the training set

TMA Name	Satisfactory QC				Suboptimal QC			
	N	AUC (95% CI)	Observed agreement (95% CI)	Kappa (95% CI)	*N*	AUC (95% CI)	Observed agreement (95% CI)	Kappa (95% CI)
TMA 1	65	82 (71, 92)	78 (67, 88)	0.31 (0.20, 0.43)	37	79 (64, 94)	84 (68, 94)	0.42 (0.25, 0.58)
TMA 2	63	93 (85, 100)	91 (82, 97)	0.78 (0.66, 0.87)	26	88 (74, 100)	86 (65, 96)	0.61 (0.41, 0.79)
TMA 3	73	92 (86, 98)	87 (76, 93)	0.61 (0.50, 0.73)	47	82 (69, 95)	79 (64, 89)	0.28 (0.17, 0.44)
TMA 4	98	86 (79, 93)	90 (83, 96)	0.69 (0.59, 0.78)	56	80 (67, 93)	82 (70, 91)	0.34 (0.25, 0.81)
TMA 5	76	91 (84, 97)	90 (80, 95)	0.70 (0.60, 0.81)	13	69 (37, 100)	89 (64, 100)	0.51 (0.60, 0.81)
TMA 6	61	89 (77, 100)	85 (74, 93)	0.49 (0.36, 0.62)	13	58 (14, 100)	77 (46, 95)	0.19 (0.10, 0.54)
TMA 7	84	88 (81, 95)	91 (82, 96)	0.69 (0.58, 0.79)	17	79 (48, 100)	88 (64, 99)	0.57 (0.33, 0.81)
TMA 8	87	89 (81, 97)	80 (71, 88)	0.38 (0.28, 0.49)	17	99 (95, 100)	78 (50, 93)	0.31 (0.10, 0.56)
TMA 9	44	100 (99, 100)	95 (85, 99)	0.85 (0.70, 0.93)	26	96 (91, 100)	95 (80, 100)	0.79 (0.61, 0.93)
TMA 10	48	98 (95, 100)	96 (86, 99)	0.88 (0.75, 0.95)	22	82 (63, 100)	95 (77, 100)	0.82 (0.60, 0.95)
TMA 11	48	92 (84, 99)	93 (83, 99)	0.81 (0.67, 0.91)	21	91 (79, 100)	85 (64, 97)	0.54 (0.30, 0.74)
TMA 12	53	93 (86, 100)	89 (77, 96)	0.55 (0.40, 0.68)	33	83 (65, 100)	87 (72, 97)	0.30 (0.16, 0.48)
TMA 13	45	86 (73, 99)	89 (76, 96)	0.68 (0.51, 0.80)	27	97 (91, 100)	96 (81, 100)	0.85 (0.66, 0.95)
TMA 14	55	89 (78, 100)	91 (80, 97)	0.75 (0.61, 0.85)	20	69 (44, 93)	76 (51, 91)	0.27 (0.11, 0.54)
TMA 15	50	91 (82, 100)	90 (78, 97)	0.71 (0.58, 0.84)	21	49 (20, 78)	78 (53, 92)	0.03 (0.01, 0.23)
Overall	950	86 (84, 89)	89 (86, 91)	0.68 (0.65, 0.71)	396	82 (78, 86)	85 (81, 88)	0.51 (0.46, 0.56)

Suboptimal QC were cores which did not meet the criteria to be considered satisfactory but which were sufficiently suitable for scoring, eg, cores with few tumour cells (50–500 cells), partially folded cores, staining artefact or suboptimal/poor fixation. *N*, Represents the number of cores on each TMA that have been classified as being either of satisfactory or suboptimal QC.

The agreement between automated and visual methods was observed to differ by the numbers of nuclei counted by the machine, with significant evidence for a positive linear correlation between mean total nuclei count and agreement parameters including kappa (*r* = 0.85; *p* = 0.004), observed agreement (*r* = 0.80; *p* = 0.01) and discriminatory accuracy (*r* = 0.76; *p* = 0.01). Kappa agreement values were highest among cores with total nuclei count >4,000–4,500 (kappa = 0.78) and least among cores with total nuclei count 50–500 (kappa = 0.41; *p*‐value for comparison = 0.01) (Table 4 and supplementary material, Figure S3). Discrepancies in extreme categories between visual and automated scores categorized in quartiles were not very common overall (∼1.3% of the cores) and this varied according to TMA as well (range = 0–4%) (supplementary material, Table S5).

**Table 4:** Agreement (observed agreement, kappa statistics) and discriminatory accuracy (AUC) parameters for automated and visual scores according to categories of the total nuclei counted by the machine among the 15 TMAs in the training set (*N* = 1,346)

Total nuclei count	*N*	AUC (95% CI)	Observed agreement (95%CI)	Kappa (95% CI)
50–500	151	80 (73, 87)	78 (71, 84)	0.41 (0.33, 0.49)
>500–1,000	227	80 (74, 86)	86 (81, 91)	0.57 (0.51, 0.64)
>1,000–1,500	207	85 (80, 90)	87 (82, 91)	0.61 (0.54, 0.68)
>1,500–2,000	172	90 (85, 95)	90 (85, 94)	0.72 (0.65, 0.79)
>2,000–2,500	106	88 (82, 95)	91 (83, 95)	0.72 (0.62, 0.80)
>2,500–3,000	87	82 (72, 92)	89 (81, 95)	0.67 (0.56, 0.76)
>3,000–3,500	90	88 (81, 95)	88 (79, 94)	0.67 (0.57, 0.77)
>3,500–4,000	74	92 (86, 98)	93 (85, 98)	0.77 (0.66, 0.86)
>4,000–4,500	56	91 (83, 99)	92 (80, 97)	0.78 (0.66, 0.88)
> 4,500	176	90 (85, 95)	88 (82, 92)	0.68 (0.61, 0.75)

N.B: Evidence for a strongly positive linear relationship between mean total nuclei count and agreement parameters was observed [kappa (*r* = 0.85, *p*‐value = 0.004); observed agreement (*r* = 0.80, *p*‐value = 0.01); AUC (*r* = 0.79, *p*‐value = 0.01)]. *N,* Represents the number of cores for each category of total nuclei count.

### Distribution of Ki67 scores by method of scoring (CAV, TMA‐specific, Universal classifier) among the 15 TMAs in the training set (*N* = 1,346 cores)

**Figure 5:**
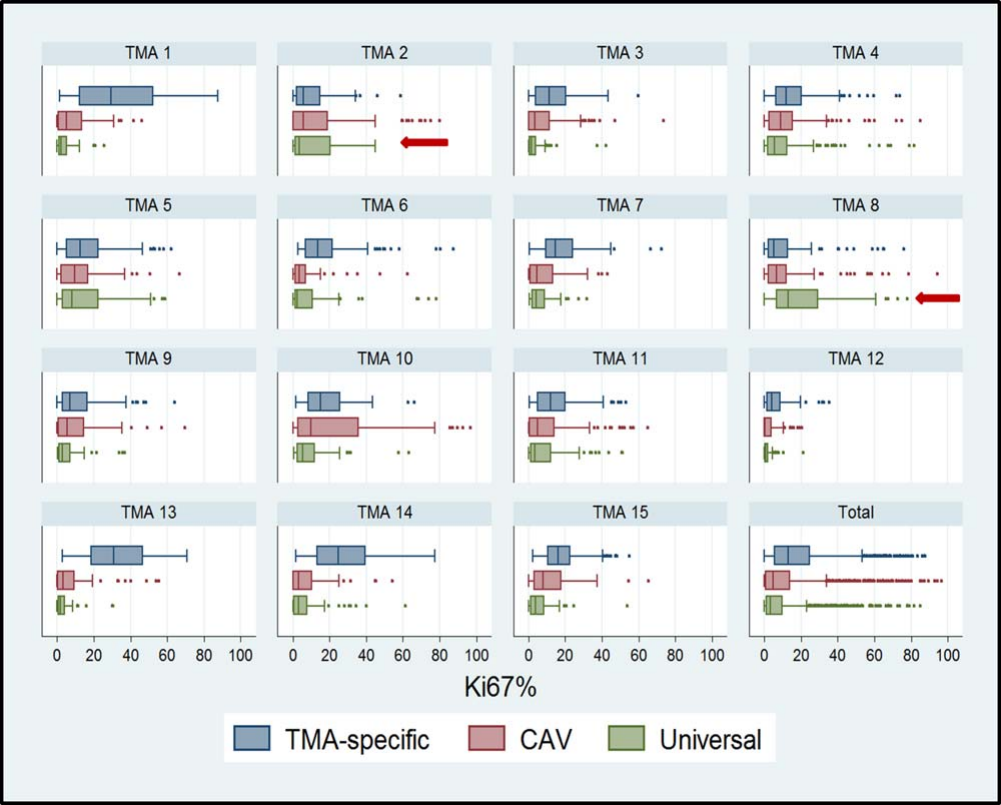
Distribution of Ki67 scores by method of scoring. Ki67 scores for the Computer‐Assisted Visual (CAV) and automated (TMA‐specific and Universal classifier) methods for each of the 15 TMAs in the training set and overall. The TMA‐specific classifier yielded higher Ki67 scores in all but two TMAs, ie, TMAs 2 and 8 (red arrows).

The TMA‐specific classifier yielded higher Ki67 values (mean = 17.5%; median = 12.9%; range = 0–85.9%) than the CAV (mean = 11.2%; median = 5.3%; range 0–96.7%) or the Universal classifier (mean = 8.8%; median = 3.7%; range = 0–84.9%) overall and in all but two of the TMAs (ie, TMAs 2 and 8) (Figure 5). Generally, the Universal classifier was tuned to count more cells than the individual TMA‐specific classifiers; this leads to a reduction in the proportion of positive relative to negative nuclei counts and hence lower Ki67 scores. As a result, the observation of lower Ki67 scores for the Universal classifier was not unexpected. In TMA 2, the Universal classifier counted fewer nuclei (supplementary material, Figure S4) than the corresponding TMA‐specific classifier and this was due to higher parameter values for axis‐ratio in the Universal relative to the TMA‐specific classifier. Lower spot width and width values for the negative relative to positive nuclei in TMA‐specific classifier 8 meant that, despite counting fewer nuclei than the Universal classifier, the TMA‐specific classifier 8 counted more negative relative to positive nuclei than the Universal classifier thereby leading to lower Ki67 scores (supplementary material, Table S4).

### Agreement between automated and visual Ki67 scores according to tumour morphology and study group for a subset of cases with visual and automated scores (*N* = 1,849 cases)

We observed better kappa agreement between the automated and visual Ki67 scores among invasive ductal (observed agreement = 90%; kappa = 0.65) than lobular (observed agreement = 86%; kappa = 0.46; *p* value for comparison = 0.003) carcinomas. Among the four study groups with visual quantitative scores in addition to automated scores, we observed good discriminatory accuracy (AUC (95% CI) = 90.0% (88–91%)) and good kappa agreement (agreement = 88.0%; kappa = 0.65) between the automated and visual scores overall. This however differed by study, with the ESTHER study showing better agreement parameters (AUC = 95%; agreement = 92%; kappa = 0.69) than the others (Table 5). It is not immediately clear what is responsible for the observed heterogeneity according to study groups given that all but one of these studies had TMA's in the training set. Indeed, when we stratified the analyses according to whether or not a study had TMAs in the training set we observed similar agreement parameters among those with TMAs in the training set (AUC = 90%; agreement = 87%; kappa = 0.54) and those without (AUC = 89%; agreement = 89%; kappa = 0.50; *p* value for comparison = 0.29) (Table 5). These findings suggest that the absence of TMAs as part of the training set from which a classifier was developed does not lead to significant attenuation of the performance of the automated methods in such TMAs.

**Table 5:** Subject level AUC and kappa agreement between automated Ki67 and visually derived scores for a subset of the participating studies for which visual scores were available (*N* = 1,849)

Study	Cases (*N*)	AUC (95% CI)	Observed agreement (95% CI)	Kappa
ABCS	215	86 (79, 94)	87 (82, 87)	0.52 (0.45, 0.59)
CNIO	154	87 (78, 97)	79 (72, 85)	0.39 (0.32, 0.47)
ESTHER	244	95 (93, 98)	92 (88, 95)	0.69 (0.62, 0.74)
PBCS	1,236	88 (87, 91)	89 (87, 91)	0.50 (0.47, 0.52)
**TMA in training set** *				
Yes	613	90 (86, 93)	87 (84, 90)	0.54 (0.50, 0.58)
No	1,236	89 (87, 91)	89 (87, 91)	0.50 (0.47, 0.52)
**Overall**	1,849	90 (88, 91)	88 (87, 90)	0.65 (0.63, 0.67)

Semi‐quantitative categories of visual scores were used to determine kappa agreement. AUC was determined using continuous automated scores and dichotomous categories of visual scores.

*Agreement analyses were stratified by whether or not a study had TMAs in the training set. ABCS, CNIO and ESTHER all had TMAs in the training set while PBCS did not have TMAs in the training set.

### Distribution of automated Ki67 scores by study group and its association with other clinical and pathological characteristics among 9,059 patients

Overall, Ki67 values differed according to the different study groups (*p*‐value <0.05) and this difference was observed when we further stratified the analysis according to whether or not TMAs were stained at the ICR; and between studies that were stained at the ICR and those that were stained externally (supplementary material, Figure S5). Analysis of histological grade as a proxy for Ki67 showed similar patterns of heterogeneity (*p*‐value <0.05). All clinical and pathological variables were seen to be significantly associated with Ki67 in logistic regression models adjusted for study group. As seen in Table 6, and as is well‐established for visual Ki67 scores, we observed strong evidence for a positive correlation between automated Ki67 and histological grade. Similarly, we observed an inverse relationship between automated Ki67 and ER and PR status. Relative to ductal carcinomas, lobular cancers were less likely to be high proliferating. The associations between HER2, EGFR and Ki67 are yet to be fully understood. In this analysis, we observed strong evidence for a positive correlation between Ki67 and HER2, CK5/6 and EGFR (Table 6).

**Table 6:** Odds ratio and 95% CI for the association between clinical and pathological characteristics of breast cancer with categories of Ki67 (≤10% vs. >10%) among 9,059 patients

Characteristic	Cases (*N*)	OR* (95% CI)	*p*‐value
**Age at diagnosis**			
<35	328	1.00 (Referent)	
35–50	3,043	0.64 (0.50–0.83)	1.00E‐03
>50–65	4,064	0.55 (0.43–0.72)	4.79E‐06
>65	1,414	0.60 (0.45–0.80)	2.43E‐04
**Tumour grade**			
Low grade	1,696	1.00 (Referent)	
Intermediate grade	3,684	1.69 (1.45–1.97)	4.71E‐12
High grade	2,552	4.18 (3.57–4.89)	3.57E‐72
**Stage**			
I	3,214	1.00 (Referent)	
II	3,534	1.15 (1.03–1.27)	1.00E‐02
III	473	1.41 (1.13–1.28)	2.00E‐03
IV	97	1.77 (1.15–2.72)	9.00E‐03
**Morphology**			
Ductal	4,315	1.00 (Referent)	
Lobular	860	0.36 (0.29–0.43)	1.98E‐25
Other	648	0.68 (0.56–0.82)	4.62E‐05
**Tumour size**			
<2 cm	4,492	1.00 (Referent)	
2–4.9 cm	2,565	1.31 (1.17–1.46)	6.64E‐07
>5 cm	244	1.29 (0.96–1.72)	8.60E‐02
**Node status**			
Negative	4,758	1.00 (Referent)	
Positive	3,168	1.11 (1.00–1.23)	4.00E‐02
**ER expression**			
Negative	2,222	1.00 (Referent)	
Positive	6,128	0.42 (0.38–0.47)	1.09E‐55
**PR expression**			
Negative	2,853	1.00 (Referent)	
Positive	4,919	0.51 (0.46–0.56)	1.68E‐36
**HER2 expression**			
Negative	5,379	1.00 (Referent)	
Positive	1,060	1.61 (1.40–1.85)	1.30E‐11
**EGFR expression**			
Negative	2,407	1.00 (Referent)	
Positive	356	3.08 (2.40–3.95)	4.61E‐19
**CK5/6 expression**			
Negative	4,184	1.00 (Referent)	
Positive	623	1.73 (1.45–2.07)	5.69E‐10

All variables were modelled separately and each model was adjusted for age at diagnosis and study group. Other morphology includes all other histological subtypes of breast cancer that are neither invasive ductal (NOS) nor invasive lobular.

*OR refers to the odds of each clinico‐pathological characteristic being high Ki67 expressing

## Discussion

This large‐scale study indicates that the Ariol automated method for high‐throughput Ki67 scoring shows good agreement with visual reads in breast cancer TMAs from multiple study populations. These findings are relevant to epidemiological research, where studies often require very large sample sizes and TMAs are frequently used to facilitate tumour characterization.

The overall agreement between the automated method and visual reads across the 166 TMAs in our study (kappa = 0.64) was within the range of kappa values previously reported by Konsti *et al* 26 (kappa = 0.57) and Mohammed *et al* 27 (kappa = 0.70). Our study however, is six times larger than the largest previously published report (Konsti, *N* = 1,334 cases), and includes multiple studies from different populations.

Some important considerations in the application of automated methods to the unsupervised scoring of Ki67 in TMAs from multiple studies are those of classifier type and the impact of core and TMA quality on the performance of these methods across the different TMAs. Compared to the Universal classifier, the TMA‐specific classifier is more time consuming, may introduce additional sources of variability, and makes comparison of results across different TMAs and/or study groups difficult to achieve. In this study, using a single Universal classifier produced similar agreement with visual scores as when using TMA‐specific classifiers. Therefore, our findings do not support any advantages of TMA‐specific over Universal classifiers.

As previously reported 18, we observed heterogeneity in the performance of the automated methods across TMAs, particularly when the TMA‐specific classifier was used. TMAs with the worst agreement parameters tended to have the highest number of cores with suboptimal QC. Discrepancies in extreme categories between visual and automated scores categorized in quartiles were not very common overall (∼1.3% of the cores). Almost all instances of such discrepancies were the direct result of poor core quality. While staining quality (background, membrane and cytoplasmic staining) was the main cause of high automated scores for cores with low visual scores, low automated scores for cores with high visual scores were mainly due to the presence of negative cell populations (such as marked lymphocytic infiltration and dense stromal components) (supplementary material, Figures S6 and S7, respectively). These reasons were also proposed to explain discrepancies in other studies 26, 29. In this study, we have also shown the impact of tissue sufficiency, using total nuclei counted by the machine as a surrogate, on the performance of the automated method. Our findings reveal that below 500 cells the performance of the automated method becomes greatly attenuated.

The analyses of the distribution of Ki67 scores among categories of other clinical and pathological characteristics showed similar patterns to those that have been previously described 24, 35, 36, 37, 38, 39, 40, 41, 42, 43. As expected, higher levels of Ki67 were strongly associated with higher histological grade 44, 45, and with ER/PR negative status 40, 41, 42, 45. Furthermore, in keeping with what is widely reported as the low proliferative activity of lobular carcinoma relative to invasive ductal carcinoma 46, 47, 48, lobular carcinomas had significantly lower Ki67 scores than invasive ductal carcinomas in this study. Our study provides strong evidence in support of a positive relationship between HER2 status and Ki67, which had been long suspected 38, 39, 45. Regarding basal markers, while the reported association between EGFR and Ki67 is largely conflicting 38, 45, 49, 50, 51, 52, that between Ki67 and CK5/6 is seldom reported. In this study, we observed higher rates of EGFR and CK5/6 positivity among high Ki67 expressing tumours, providing the most definitive evidence to date in support of these associations. The evidence for a relationship between Ki67 and nodal status is not conclusive despite this being one of the most studied aspects of Ki67. In a review by Urruticoechea and colleagues 45, while a few large studies (>200 patients) were reported to show a positive relationship between Ki67 and nodal status 53, 54, 55, numerous small ones favoured a lack of correlation 45. Our findings support a positive correlation between Ki67 and nodal status.

A major strength of this study is its large size, detailed information on pathology variables, and the inclusion of TMAs from diverse populations conducted in different time periods, reflecting a likely scenario in epidemiological pooling studies. Our algorithm was validated against quantitative visual scores derived using the CAV protocol. Additionally, the algorithm performed well against other methods of manually counting Ki67 other than the CAV method thereby providing additional validation for the automated method.

Stringent pre and post analytical QC protocols were applied to the generation of Ki67 scores. While this improves the performance of automated scoring, it also reduces its comparative advantage by being more time consuming. Furthermore, although good agreement was observed between the automated and the CAV scores, misclassification of malignant as benign ductal epithelial or stromal cells and/or positively staining as negatively staining malignant cells is likely to lead to the underestimation of relationships between Ki67 and other pathology markers, risk factors and/or survival outcomes. Misclassification can result from a number of factors including the inability of the automated methods to distinguish between benign and malignant epithelial cells and quality control issues. Future work is thus needed to improve the detection of cancer cells by automated methods, and to develop automated measures of quality control, such as total nuclear count, intensity values, proportion of poor quality cores/TMA etc. For instance, we observed that TMA spots with extremely low total nuclei counts (<50) were mostly those in which no tissue core was present; that those with Ki67 scores of exactly 100% were mostly those with staining problems; and that those with spuriously high total nuclei counts (>15,000) were mostly lymph nodes showing occasional metastatic foci of malignant cells. Based on these observations, we believe that ‘automation‐derived quality control indices’ can be developed and, if validated, used a priori for the definition of core/TMA exclusion and/or inclusion criteria. Lastly, even though the Ariol system is not widely available, increasing compatibility between platforms coupled with the gradual rise in the number of open source software should allow for the application of automated systems on a wider scale 29, 56.

In conclusion, investigating aetiological and prognostic heterogeneity among IHC defined subtypes of breast cancer requires the incorporation of measures of Ki67 and other IHC markers in large‐scale collaborative molecular epidemiological studies. Even though high‐throughput and reproducible, concerns remain about the accuracy of automated methods and the quality of the data derived when such methods are used on a large‐scale. Here, we have shown that when applied to the large‐scale scoring of Ki67 in breast cancer TMAs from different populations, automated systems constitute highly efficient methods for generating good quality data. However, concerted efforts at algorithm development together with rigorous pre‐analytical quality control processes are necessary to ensure satisfactory performance.

## Author contributions

MA and MG‐C conceived and carried out the analysis; MG‐C supervised the work; FD and LZ carried out centralised laboratory work; MA developed the CAV and automated scoring protocols with additional support from LZ (CAV), FD, LZ, WJH and L‐AMcD (automated protocol); NO, AJS and MD provided additional supervisory support; PC performed data management; MA, MG‐C analysed the data; FB, HRA, PC, JB, RM, HB, CS, AM, JCC, AR, PS, FJC, REAMT, PD, JF, MES, JL, DE, MJH, AH, JWMM, CHMvD, MKB, QW, MJ, MS, AJS, DE, AB, LV‘tV, FEvL, MKS, PDP contributed to TMA/data collection and/or data management. All authors contributed to manuscript development and writing and gave final approval for its submission.

## Supporting information

SUPPLEMENTARY MATERIAL ONLINE

This supplementary file contains Tables S1 to S5:
**Table S1.** Immunohistochemistry reagents and antigen retrieval protocols
**Table S2.** Core (*N*=202) and subject (*N*=101) level inter‐rater agreement and agreement between the CAV protocol and each scorer with the Ariol automated quantitative Ki67 scores
**Table S3.** Colour parameters (hue, saturation, intensity) for distinguishing negative (haematoxylin) and positive (DAB) nuclei using the Ariol automated scoring algorithm for TMA‐specific classifiers – TMA 1–15, and Universal classifier
**Table S4.** Shape parameters (spot width, width, compactness, roundness and axis ratio) for distinguishing negative (haematoxylin) from positive (DAB) nuclei using the Ariol automated scoring algorithm for TMA‐specific classifiers – TMA 1–15, and Universal classifier
**Table S5.** Cross‐tabulation of visual and automated Ki67 scores (TMA's 1–15 and overall)

**Figure S1 (TMAs 1–15 & overall).** Graphs comparing the ROC curves for the discriminatory accuracy of the automated continuous Ki67 scores against categories of the visual score by classifier type (TMA‐specific and universal) among each of the 15 TMAs in the training set and overall

**Figure S2 (TMAs 1–15 & overall).** Graphs comparing the ROC curves for the discriminatory accuracy of the automated continuous scores against categories of the visual score by QC status among all 15 TMAs in the training set and overall

**Figure S3.** ROC curves, by total nuclei count, for the discriminatory accuracy of the automated quantitative Ki67 scores against categories of the visual score

**Figure S4.** Distribution of total nuclei counted by the machine for the TMA‐specific and universal classifiers among the 15 TMAs in the training set and overall

**Figure S5.** Distribution of the subject level (*N*=9,059) Ki67 score among (A) the different study groups, (B) according to whether the TMAs were stained at the ICR or in an external location, (C) among study groups whose TMAs were stained at the ICR and (D) among study groups whose TMAs were stained in an external location

**Figure S6.** Screengrab for a representative core in which discrepancy (ie, visual category 1 and Ariol category 4) between visual and automated scores was observed. The most common causes of ‘false positive’ by the machine are related to quality control: more specifically, the presence of background staining, core folding and membrane (instead of nuclear) staining. Of these, membrane staining was more prevalent and was observed in 8.7% of the cores

**Figure S7.** Screengrab for a representative core in which discrepancy (ie, visual category 4 and Ariol category 1) between visual and automated scores was observed. The most common causes of ‘false negatives’ by the machine include marked lymphocytic infiltration with only occasional nests of invasive malignant cells, poor fixation, nuclear halo, and very low intensity DAB
