# Supplementary material for: High‐throughput automated scoring of Ki67 in breast cancer tissue microarrays from the Breast Cancer Association Consortium
Source: J Pathol Clin Res. 2016 Apr 6;2(3):138–53. doi: 10.1002/cjp2.42 (PMC4958735; doi:10.1002/cjp2.42)
Supplement: Supplementary file 1 — This supplementary file contains Tables S1 to S5: Table S1. Immunohistochemistry reagents and antigen retrieval protocols Table S2. Core (N=202) and subject (N=101) level inter‐rater agreement and agreement between the CAV protocol and each scorer with the Ariol automated quantitative Ki67 scores Table S3. Colour parameters (hue, saturation, intensity) for distinguishing negative (haematoxylin) and positive (DAB) nuclei using the Ariol automated scoring algorithm for TMA‐specific classifiers – TMA 1–15, and Universal classifier Table S4. Shape parameters (spot width, width, compactness, roundness and axis ratio) for distinguishing negative (haematoxylin) from positive (DAB) nuclei using the Ariol automated scoring algorithm for TMA‐specific classifiers – TMA 1–15, and Universal classifier Table S5. Cross‐tabulation of visual and automated Ki67 scores (TMA's 1–15 and overall) [file CJP2-2-138-s008.docx]

**Supplementary Table 1:** Ki67 immunohistochemistry reagents and antigen retrieval protocols according to study groups

| **Study** | **Clone** | **Clonality** | **Source** | **Dilution** | **Antigen**  **retrieval** |
| --- | --- | --- | --- | --- | --- |
|  |  |  |  |  |  |
| PBCS | MIB-1 | Mouse monoclonal | DAKO | 1:500 | Tris-EDTA buffer,  pH9, 20 minutes |
|  |  |  |  |  |  |
| SEARCH | MIB-1 | Mouse monoclonal | DAKO | 1:200 | Tris-EDTA buffer,  pH9, 30 minutes |
|  |  |  |  |  |  |
| MARIE | MIB-1 | Mouse monoclonal | DAKO | 1:400 | Tris-EDTA buffer,  pH9, 10 minutes |

**Supplementary Table 2**: Core (N = 202) and subject (N = 101) level inter-rater agreement and agreement between the CAV protocol and each scorer with the Ariol automated quantitative Ki67 scores.

|  |  | **Core-level**  **agreement** |  |  |
| --- | --- | --- | --- | --- |
|  |  |  |  |  |
| Comparison | **N** | **AUC**  **(95%CI)** | **Observed**  **agreement (95%CI)** | **Kappa(95%CI)** |
| CAV vs. scorer 2 | 202 | 91 (87, 96) | 87 (82, 91) | 0.66 (0.59, 0.72) |
| CAV vs. scorer 3 | 202 | 87 (81, 92) | 84 (78, 89) | 0.59 (0.52, 0.66) |
| scorer 2 vs. scorer 3 | 202 | - | 89 (84, 93) | 0.69 (0.62, 0.75) |
| Ariol vs. CAV | 202 | 95 (92, 98) | 87 (82, 91) | 0.69 (0.62, 0.76) |
| Ariol vs. scorer 2 | 202 | 88 (84, 93) | 82 (76, 87) | 0.54 (0.46, 0.60) |
| Ariol vs. scorer 3 | 202 | 83 (77, 89) | 80 (74, 85) | 0.46 (0.39, 0.53) |
|  |  |  |  |  |
|  |  | **Subject-level**  **agreement** |  |  |
|  |  |  |  |  |
| Comparison | **N** | **AUC**  **(95%CI)** | **Observed**  **agreement (95%CI)** | **Kappa(95%CI)** |
| CAV vs. scorer 2 | 101 | 97 (93, 100) | 92 (85, 97) | 0.79 (0.70, 0.87) |
| CAV vs. scorer 3 | 101 | 96 (91, 100) | 89 (81, 94) | 0.69 (0.59, 0.77) |
| scorer 2 vs. scorer 3 | 101 | - | 90 (83, 95) | 0.67 (0.58, 0.77) |
| Ariol vs. CAV | 101 | 96 (92, 99) | 87 (79, 93) | 0.69 (0.59, 0.77) |
| Ariol vs. scorer 2 | 101 | 94 (87, 100) | 88 (80, 94) | 0.69 (0.60, 0.78) |
| Ariol vs. scorer 3 | 101 | 88 (79, 97) | 86 (78, 92) | 0.62 (0.52, 0.71) |

CAV = Computer-Assisted Visual scoring protocol performed by one of the pathologists (MA).

Ki67 scores by scorers 1 & 2 are semi-quantitative i.e. 0, 25%, 50%, 75% or 100%.

CAV and Ariol are quantitative scores (0 – 100%).

Weighted kappa was determined using semi-quantitative categories of scores from CAV, scorer 2, scorer 3, and Ariol.

The AUC was determined plotting continuous scores for CAV and Ariol against dichotomous categories of scores by scorer 2 and 3 (dichotomised at 25% cut-off point).

Each individual had two cores; as such, subject level data represent the average score from both cores.

**Supplementary Table 3**: Colour parameters (hue, saturation, intensity) for distinguishing negative (haematoxylin) and positive (DAB) nuclei using the Ariol automated scoring algorithm for TMA-specific classifiers – TMA 1 to 15, and Universal classifier.

|  | **Haematoxylin/Negative nuclei** | | | **DAB/Positive nuclei** | | |
| --- | --- | --- | --- | --- | --- | --- |
| **Classifier** | **Hue*** | **Saturation** | **Intensity** | **Hue*** | **Saturation** | **Intensity** |
| **TMA-specific** |  |  |  |  |  |  |
| TMA 1 | 45/84 | 17/42 | 152/201 | 82/140 | 23/123 | 56/169 |
| TMA 2 | 29/115 | 14/82 | 88/200 | 121/152 | 20/147 | 38/207 |
| TMA 3 | 29/115 | 14/82 | 88/200 | 121/152 | 15/147 | 38/207 |
| TMA 4 | 45/116 | 9/82 | 88/207 | 117/146 | 19/147 | 38/190 |
| TMA 5 | 48/102 | 8/37 | 141/211 | 121/149 | 20/103 | 85/183 |
| TMA 6 | 51/107 | 10/47 | 154/210 | 99/154 | 19/124 | 51/197 |
| TMA 7 | 50/106 | 11/45 | 155/211 | 98/156 | 19/124 | 51/197 |
| TMA 8 | 38/117 | 9/33 | 157/209 | 122/159 | 14/165 | 31/194 |
| TMA 9 | 48/104 | 15/49 | 128/200 | 117/152 | 24/147 | 38/196 |
| TMA 10 | 48/104 | 15/48 | 128/200 | 116/152 | 24/182 | 21/196 |
| TMA 11 | 48/104 | 15/49 | 128/200 | 112/152 | 19/182 | 22/197 |
| TMA 12 | 48/104 | 15/49 | 128/200 | 115/152 | 14/182 | 21/196 |
| TMA 13 | 48/104 | 15/49 | 128/200 | 115/152 | 15/182 | 21/196 |
| TMA 14 | 50/104 | 22/49 | 131/200 | 115/152 | 14/182 | 21/196 |
| TMA 15 | 49/93 | 17/37 | 150/198 | 117/145 | 12/173 | 26/202 |
|  |  |  |  |  |  |  |
| **Universal** | 50/89 | 11/58 | 126/198 | 96/147 | 12/147 | 38/191 |

N.B: Values represent the parameters for the upper and lower sliders of hue, saturation and intensity. The multi-stain high resolution script was used and colour 1 (pseudo – red) was tuned for negative nuclei while colour 2 (pseudo – green) was tuned for positive nuclei.

*Hue is the gradation of the selected colour pixels for Haematoxylin and DAB.

**Supplementary Table 4**: Shape parameters (spot width, width, compactness, roundness and axis ratio) for distinguishing negative (haematoxylin) from positive (DAB) nuclei using the Ariol automated scoring algorithm for TMA-specific classifiers – TMA 1 to 15, and Universal classifier.

|  | **Negative/Haematoxylin nuclei** | | | | | **DAB/Positive nuclei** | | | | |
| --- | --- | --- | --- | --- | --- | --- | --- | --- | --- | --- |
| **Classifier type** | **Spot**  **width** | **Width** | **Compactness** | **Roundness** | **Axis**  **ratio** | **Spot**  **width** | **Width** | **Compactness** | **Roundness** | **Axis**  **ratio** |
| **TMA-specific** |  |  |  |  |  |  |  |  |  |  |
| TMA 1 | 2/100 | 2/100 | 200/1000 | 200/1000 | 70/1000 | 2/100 | 2/100 | 200/1000 | 200/1000 | 70/1000 |
| TMA 2 | 2/100 | 2/100 | 200/1000 | 200/1000 | 70/1000 | 2/100 | 2/100 | 200/1000 | 200/1000 | 70/1000 |
| TMA 3 | 5/100 | 5/100 | 200/1000 | 1/1000 | 1/1000 | 2/100 | 0/100 | 1/1000 | 1/1000 | 1/1000 |
| TMA 4 | 5/100 | 5/100 | 200/1000 | 200/1000 | 1/1000 | 2/100 | 0/100 | 1/1000 | 1/1000 | 1/1000 |
| TMA 5 | 5/100 | 5/100 | 200/1000 | 200/1000 | 1/1000 | 2/100 | 2/100 | 1/1000 | 1/1000 | 1/1000 |
| TMA 6 | 5/100 | 5/100 | 200/1000 | 200/1000 | 70/1000 | 2/100 | 2/100 | 200/1000 | 200/1000 | 13/1000 |
| TMA 7 | 5/100 | 5/100 | 200/1000 | 200/1000 | 70/1000 | 2/100 | 2/100 | 200/1000 | 200/1000 | 13/1000 |
| TMA 8 | 5/14 | 4/14 | 200/1000 | 200/1000 | 301/1000 | 6/11 | 5/100 | 200/1000 | 200/1000 | 301/1000 |
| TMA 9 | 5/16 | 4/14 | 200/1000 | 200/1000 | 134/1000 | 5/14 | 4/14 | 200/1000 | 200/1000 | 134/1000 |
| TMA 10 | 3/100 | 3/100 | 200/1000 | 200/1000 | 134/1000 | 2/100 | 2/100 | 200/1000 | 200/1000 | 111/1000 |
| TMA 11 | 4/93 | 4/100 | 200/1000 | 200/1000 | 70/1000 | 3/83 | 1/100 | 200/1000 | 200/1000 | 82/1000 |
| TMA 12 | 3/93 | 4/100 | 200/1000 | 151/1000 | 70/1000 | 3/83 | 1/100 | 200/1000 | 200/1000 | 82/1000 |
| TMA 13 | 3/83 | 4/100 | 200/1000 | 203/1000 | 76/1000 | 1/80 | 1/100 | 200/1000 | 200/1000 | 82/1000 |
| TMA 14 | 3/83 | 4/100 | 200/1000 | 203/1000 | 76/1000 | 1/80 | 1/100 | 200/1000 | 200/1000 | 82/1000 |
| TMA 15 | 4/30 | 4/100 | 203/1000 | 203/1000 | 99/1000 | 4/14 | 5/100 | 203/1000 | 203/1000 | 82/1000 |
|  |  |  |  |  |  |  |  |  |  |  |
| **Universal** | 2/100 | 2/100 | 200/1000 | 200/1000 | 134/1000 | 2/100 | 2/100 | 200/1000 | 200/1000 | 134/1000 |

**Supplementary Table 5:** Cross-tabulation of visual and automated scores for all 15 TMAs in the training set and overall.

| **TMA 1** | Automated score | | | |  |  |  |
| --- | --- | --- | --- | --- | --- | --- | --- |
| Visual | 1 | 2 | 3 | 4 | Total |  |  |
| 1 | 10 | 9 | 2 | 2 | 23 |  |  |
| 2 | 16 | 8 | 3 | 2 | 29 | Observed agreement | 80% |
| 3 | 3 | 14 | 6 | 1 | 24 | Weighted kappa | 0.37 |
| 4 | 1 | 9 | 12 | 4 | 26 |  |  |
| Total | 30 | 40 | 23 | 9 | 102 |  |  |
|  |  |  |  |  |  |  |  |
| **TMA 2** |  | Automated score | | |  |  |  |
| Visual | 1 | 2 | 3 | 4 | Total |  |  |
| 1 | 11 | 10 | 0 | 1 | 22 |  |  |
| 2 | 7 | 8 | 2 | 2 | 19 | Observed agreement | 90% |
| 3 | 3 | 2 | 2 | 6 | 13 | Weighted kappa | 0.75 |
| 4 | 0 | 1 | 8 | 26 | 35 |  |  |
| Total | 21 | 21 | 12 | 35 | 89 |  |  |
|  |  |  |  |  |  |  |  |
| **TMA 3** |  | Automated score | | |  |  |  |
| Visual | 1 | 2 | 3 | 4 | Total |  |  |
| 1 | 26 | 2 | 3 | 2 | 33 |  |  |
| 2 | 18 | 12 | 2 | 0 | 32 | Observed agreement | 84% |
| 3 | 6 | 13 | 11 | 0 | 30 | Weighted kappa | 0.49 |
| 4 | 0 | 8 | 14 | 3 | 25 |  |  |
| Total | 50 | 35 | 30 | 5 | 120 |  |  |
|  |  |  |  |  |  |  |  |
| **TMA 4** |  | Automated score | | |  |  |  |
| Visual | 1 | 2 | 3 | 4 | Total |  |  |
| 1 | 7 | 10 | 5 | 0 | 22 |  |  |
| 2 | 13 | 8 | 8 | 4 | 33 | Observed agreement | 87% |
| 3 | 2 | 7 | 35 | 11 | 55 | Weighted kappa | 0.58 |
| 4 | 2 | 5 | 5 | 32 | 44 |  |  |
| Total | 24 | 30 | 53 | 47 | 154 |  |  |
|  |  |  |  |  |  |  |  |
| **TMA 5** |  | Automated score | | |  |  |  |
| Visual | 1 | 2 | 3 | 4 | Total |  |  |
| 1 | 9 | 4 | 2 | 0 | 15 |  |  |
| 2 | 1 | 6 | 7 | 5 | 19 | Observed agreement | 89% |
| 3 | 0 | 2 | 4 | 22 | 28 | Weighted kappa | 0.69 |
| 4 | 0 | 0 | 5 | 22 | 27 |  |  |
| Total | 10 | 12 | 18 | 49 | 89 |  |  |
|  |  |  |  |  |  |  |  |
| **TMA 6** |  | Automated score | | |  |  |  |
| Visual | 1 | 2 | 3 | 4 | Total |  |  |
| 1 | 7 | 3 | 2 | 3 | 15 |  |  |
| 2 | 8 | 14 | 4 | 4 | 30 | Observed agreement | 84% |
| 3 | 1 | 9 | 6 | 5 | 21 | Weighted kappa | 0.44 |
| 4 | 0 | 1 | 1 | 6 | 8 |  |  |
| Total | 16 | 27 | 13 | 18 | 74 |  |  |
|  |  |  |  |  |  |  |  |
| **TMA 7** |  | Automated score | | |  |  |  |
| Visual | 1 | 2 | 3 | 4 | Total |  |  |
| 1 | 11 | 7 | 1 | 0 | 19 |  |  |
| 2 | 4 | 17 | 12 | 5 | 38 | Observed agreement | 90% |
| 3 | 0 | 5 | 12 | 5 | 22 | Weighted kappa | 0.67 |
| 4 | 0 | 1 | 11 | 10 | 22 |  |  |
| Total | 15 | 30 | 36 | 20 | 101 |  |  |

| Table 5 cont. |  |  |  |  |  |  |  |
| --- | --- | --- | --- | --- | --- | --- | --- |
| **TMA 8** |  | Automated score | | |  |  |  |
| Visual | 1 | 2 | 3 | 4 | Total |  |  |
| 1 | 4 | 5 | 7 | 1 | 17 |  |  |
| 2 | 0 | 2 | 10 | 12 | 24 | Observed agreement | 80% |
| 3 | 1 | 2 | 8 | 29 | 40 | Weighted kappa | 0.37 |
| 4 | 0 | 0 | 2 | 21 | 23 |  |  |
| Total | 5 | 9 | 27 | 63 | 104 |  |  |
|  |  |  |  |  |  |  |  |
| **TMA 9** |  | Automated score | | |  |  |  |
| Visual | 1 | 2 | 3 | 4 | Total |  |  |
| 1 | 13 | 8 | 1 | 0 | 22 |  |  |
| 2 | 2 | 10 | 0 | 0 | 12 | Observed agreement | 95% |
| 3 | 0 | 7 | 8 | 3 | 18 | Weighted kappa | 0.85 |
| 4 | 0 | 0 | 8 | 10 | 18 |  |  |
| Total | 15 | 25 | 17 | 13 | 70 |  |  |
|  |  |  |  |  |  |  |  |
| **TMA 10** |  | Automated score | | |  |  |  |
| Visual | 1 | 2 | 3 | 4 | Total |  |  |
| 1 | 7 | 1 | 0 | 0 | 8 |  |  |
| 2 | 5 | 9 | 1 | 0 | 15 | Observed agreement | 96% |
| 3 | 0 | 2 | 11 | 3 | 16 | Weighted kappa | 0.87 |
| 4 | 0 | 1 | 10 | 20 | 31 |  |  |
| Total | 12 | 13 | 22 | 23 | 70 |  |  |
|  |  |  |  |  |  |  |  |
| **TMA 11** |  | Automated score | | |  |  |  |
| Visual | 1 | 2 | 3 | 4 | Total |  |  |
| 1 | 11 | 3 | 3 | 0 | 17 |  |  |
| 2 | 7 | 7 | 5 | 1 | 20 | Observed agreement | 90% |
| 3 | 1 | 4 | 8 | 1 | 14 | Weighted kappa | 0.73 |
| 4 | 0 | 1 | 1 | 16 | 18 |  |  |
| Total | 19 | 15 | 17 | 18 | 69 |  |  |
|  |  |  |  |  |  |  |  |
| TMA 12 |  | Automated score | | |  |  |  |
| Visual | 1 | 2 | 3 | 4 | Total |  |  |
| 1 | 37 | 10 | 1 | 1 | 49 |  |  |
| 2 | 12 | 4 | 3 | 0 | 19 | Observed agreement | 88% |
| 3 | 5 | 4 | 3 | 0 | 12 | Weighted kappa | 0.47 |
| 4 | 0 | 2 | 3 | 1 | 6 |  |  |
| Total | 54 | 20 | 10 | 2 | 86 |  |  |
|  |  |  |  |  |  |  |  |
| **TMA 13** |  | Automated score | | |  |  |  |
| Visual | 1 | 2 | 3 | 4 | Total |  |  |
| 1 | 16 | 5 | 0 | 0 | 21 |  |  |
| 2 | 6 | 13 | 3 | 0 | 22 | Observed agreement | 92% |
| 3 | 1 | 8 | 6 | 1 | 16 | Weighted kappa | 0.73 |
| 4 | 2 | 0 | 7 | 4 | 13 |  |  |
| Total | 25 | 26 | 16 | 5 | 72 |  |  |
|  |  |  |  |  |  |  |  |
| **TMA 14** |  | Automated score | | |  |  |  |
| Visual | 1 | 2 | 3 | 4 | Total |  |  |
| 1 | 18 | 3 | 2 | 1 | 24 |  |  |
| 2 | 6 | 6 | 6 | 3 | 21 | Observed agreement | 87% |
| 3 | 2 | 3 | 4 | 5 | 14 | Weighted kappa | 0.64 |
| 4 | 0 | 1 | 6 | 9 | 16 |  |  |
| Total | 26 | 13 | 18 | 18 | 75 |  |  |

| Table 5 cont. |  |  |  |  |  |  |  |
| --- | --- | --- | --- | --- | --- | --- | --- |
| **TMA 15** |  | Automated score | | |  |  |  |
| Visual | 1 | 2 | 3 | 4 | Total |  |  |
| 1 | 6 | 1 | 1 | 0 | 8 |  |  |
| 2 | 5 | 10 | 5 | 1 | 21 | Observed agreement | 87% |
| 3 | 3 | 7 | 7 | 2 | 19 | Weighted kappa | 0.56 |
| 4 | 1 | 2 | 12 | 8 | 23 |  |  |
| Total | 15 | 20 | 25 | 11 | 71 |  |  |
|  |  |  |  |  |  |  |  |
| **Overall** |  | Automated score | | |  |  |  |
| Visual | 1 | 2 | 3 | 4 | Total |  |  |
| 1 | 193 | 81 | 30 | 11 | 315 |  |  |
| 2 | 110 | 134 | 71 | 39 | 354 | Observed | 87% |
|  |  |  |  |  |  | agreement |  |
| 3 | 28 | 89 | 131 | 94 | 342 | Kappa | 0.64 |
| 4 | 6 | 32 | 105 | 192 | 335 |  |  |
| Total | 337 | 336 | 337 | 336 | 1,346 |  |  |
