# Supplementary material for: High‐throughput automated scoring of Ki67 in breast cancer tissue microarrays from the Breast Cancer Association Consortium
Source: J Pathol Clin Res. 2016 Apr 6;2(3):138–53. doi: 10.1002/cjp2.42 (PMC4958735; doi:10.1002/cjp2.42)
Supplement: Supplementary file 2 — Figure S1 (TMAs 1–15 & overall). Graphs comparing the ROC curves for the discriminatory accuracy of the automated continuous Ki67 scores against categories of the visual score by classifier type (TMA‐specific and universal) among each of the 15 TMAs in the training set and overall [file CJP2-2-138-s001.pdf]

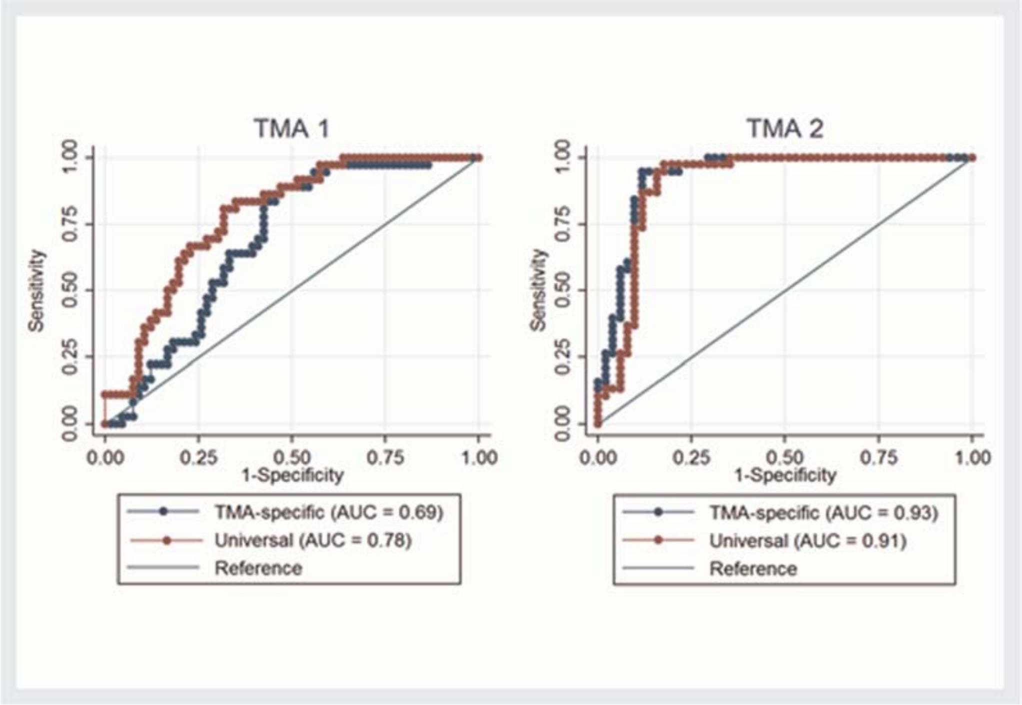

Supplementary Figure S1A (TMAs 1–15 & overall).: Graphs comparing the ROC curves for the discriminatory accuracy of the automated continuous Ki67 scores against categories of the visual score by classifier type (TMA-specific and universal) among each of the 15 TMAs in the training set and overall.

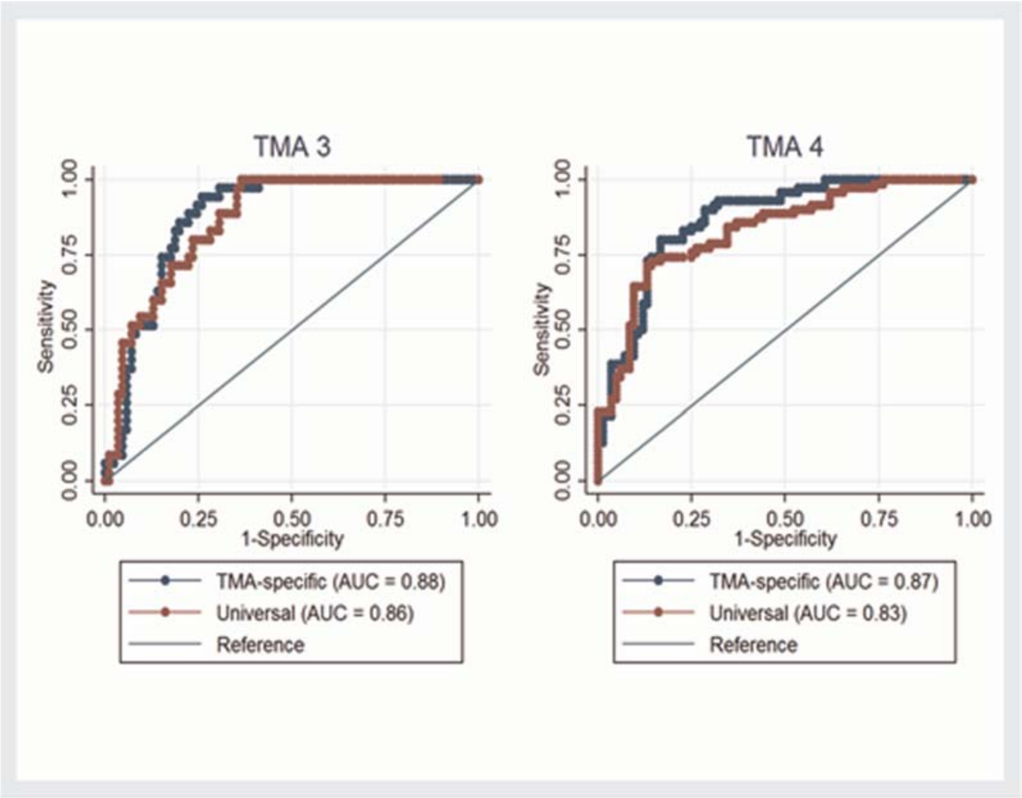

Supplementary Figure S1B (TMAs 1–15 & overall).: Graphs comparing the ROC curves for the discriminatory accuracy of the automated continuous Ki67 scores against categories of the visual score by classifier type (TMA-specific and universal) among each of the 15 TMAs in the training set and overall.

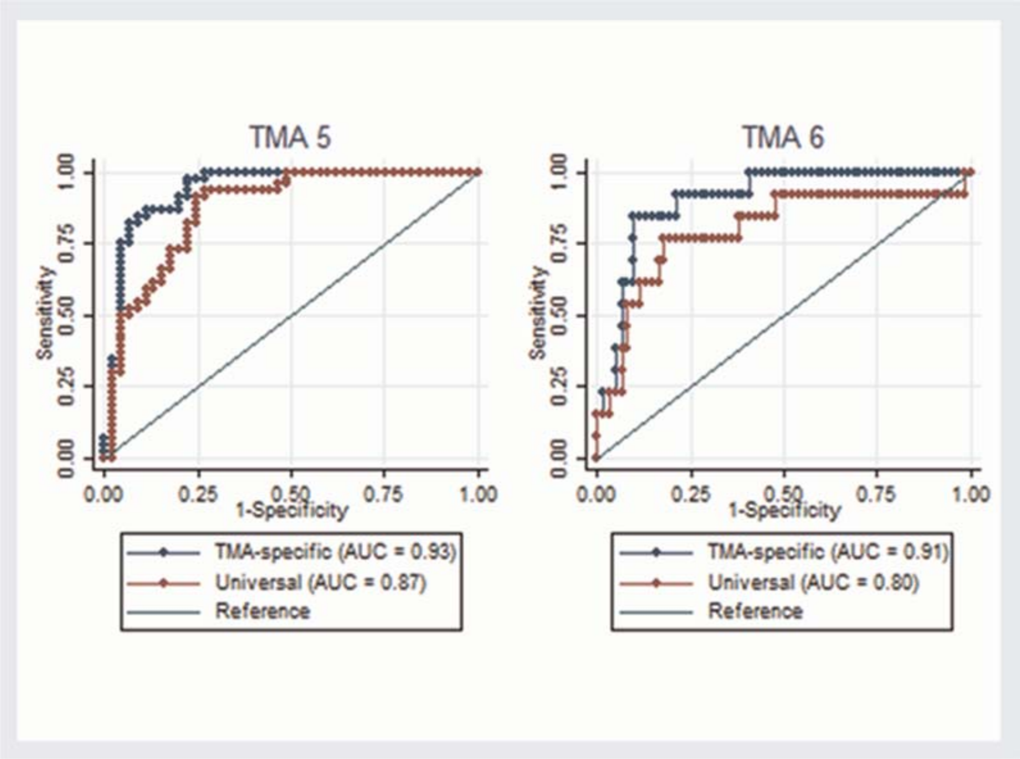

Supplementary Figure S1C (TMAs 1–15 & overall).: Graphs comparing the ROC curves for the discriminatory accuracy of the automated continuous Ki67 scores against categories of the visual score by classifier type (TMA-specific and universal) among each of the 15 TMAs in the training set and overall.

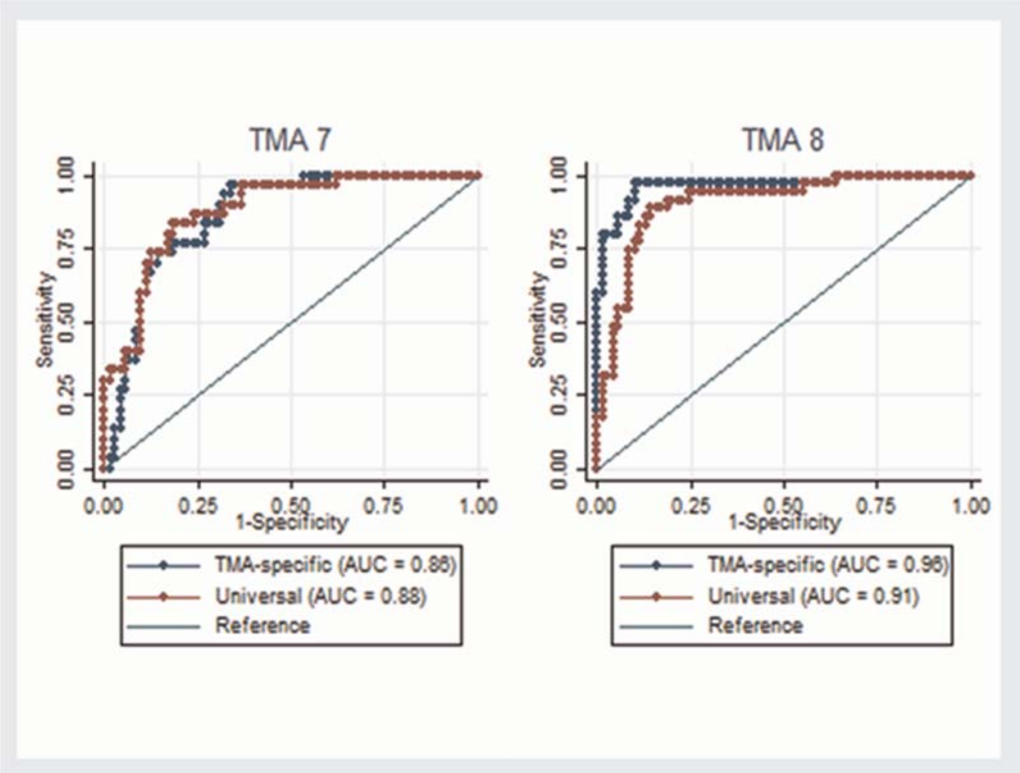

Supplementary Figure S1D (TMAs 1–15 & overall).: Graphs comparing the ROC curves for the discriminatory accuracy of the automated continuous Ki67 scores against categories of the visual score by classifier type (TMA-specific and universal) among each of the 15 TMAs in the training set and overall.

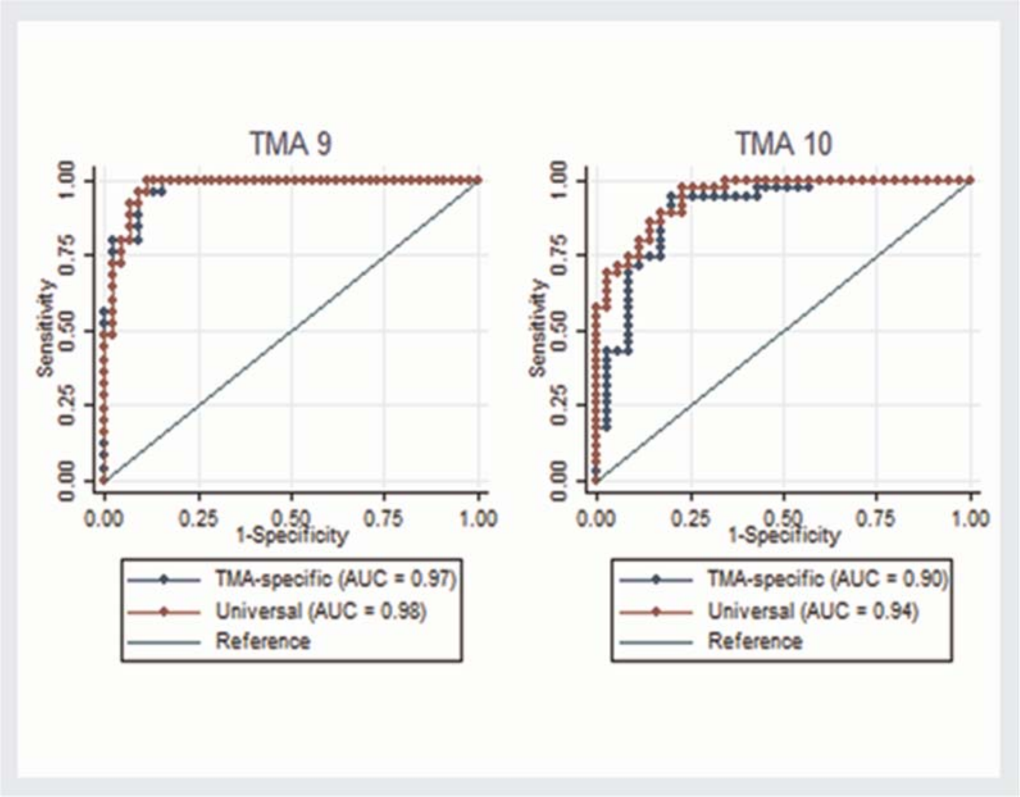

Supplementary Figure S1E (TMAs 1–15 & overall).: Graphs comparing the ROC curves for the discriminatory accuracy of the automated continuous Ki67 scores against categories of the visual score by classifier type (TMA-specific and universal) among each of the 15 TMAs in the training set and overall.

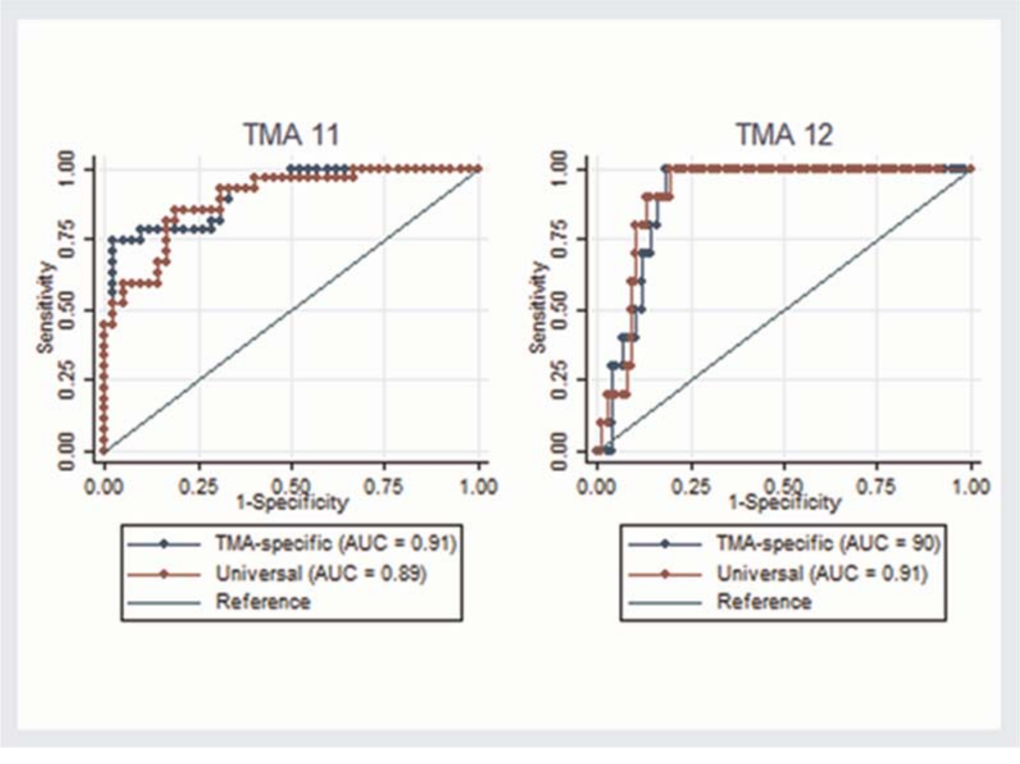

Supplementary Figure S1F (TMAs 1–15 & overall).: Graphs comparing the ROC curves for the discriminatory accuracy of the automated continuous Ki67 scores against categories of the visual score by classifier type (TMA-specific and universal) among each of the 15 TMAs in the training set and overall.

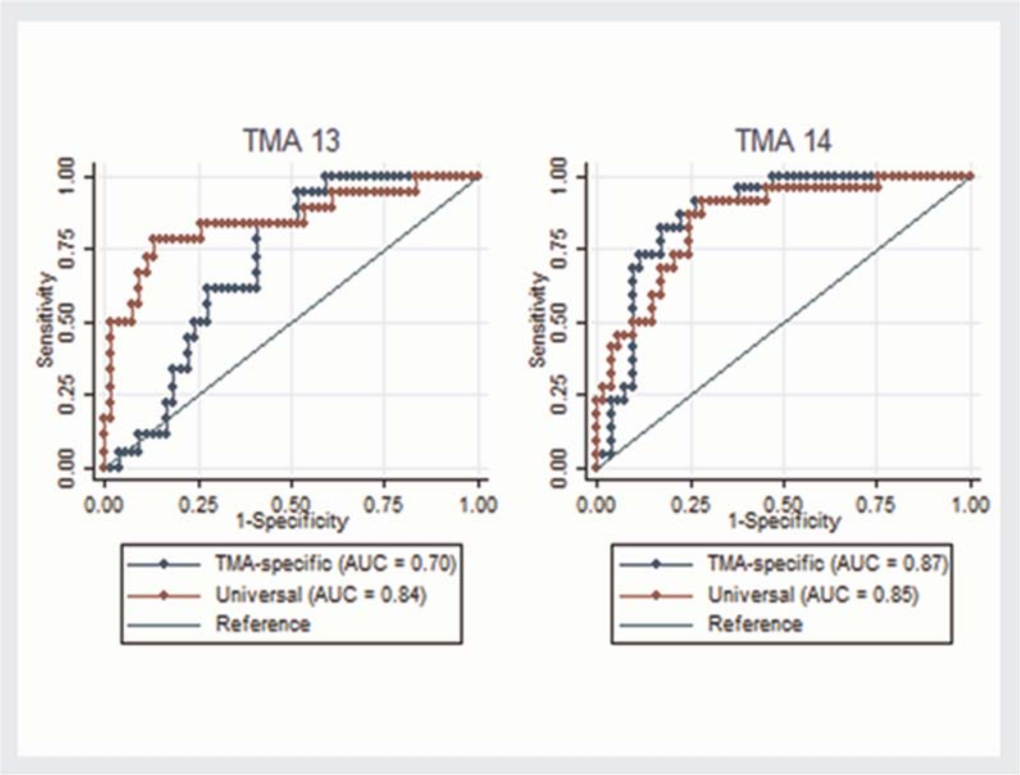

Supplementary Figure S1G (TMAs 1–15 & overall).: Graphs comparing the ROC curves for the discriminatory accuracy of the automated continuous Ki67 scores against categories of the visual score by classifier type (TMA-specific and universal) among each of the 15 TMAs in the training set and overall.

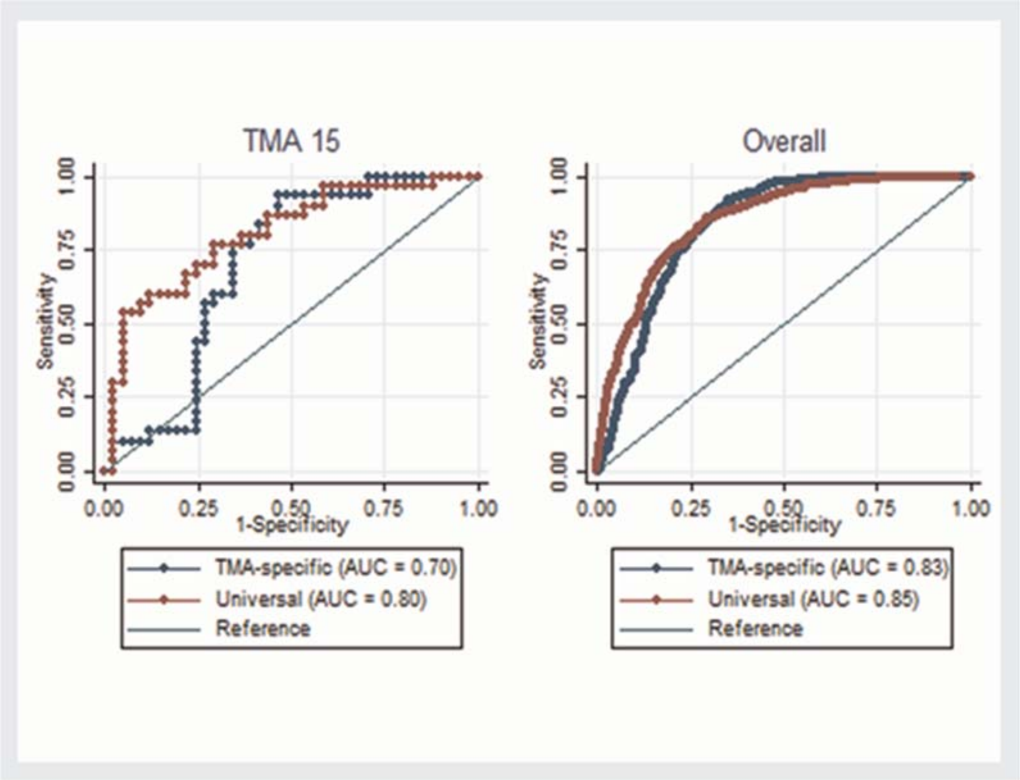

Supplementary Figure S1H (TMAs 1–15 & overall).: Graphs comparing the ROC curves for the discriminatory accuracy of the automated continuous Ki67 scores against categories of the visual score by classifier type (TMA-specific and universal) among each of the 15 TMAs in the training set and overall.
