# Supplementary material for: High‐throughput automated scoring of Ki67 in breast cancer tissue microarrays from the Breast Cancer Association Consortium
Source: J Pathol Clin Res. 2016 Apr 6;2(3):138–53. doi: 10.1002/cjp2.42 (PMC4958735; doi:10.1002/cjp2.42)
Supplement: Supplementary file 3 — Figure S2 (TMAs 1–15 & overall). Graphs comparing the ROC curves for the discriminatory accuracy of the automated continuous scores against categories of the visual score by QC status among all 15 TMAs in the training set and overall [file CJP2-2-138-s002.pdf]

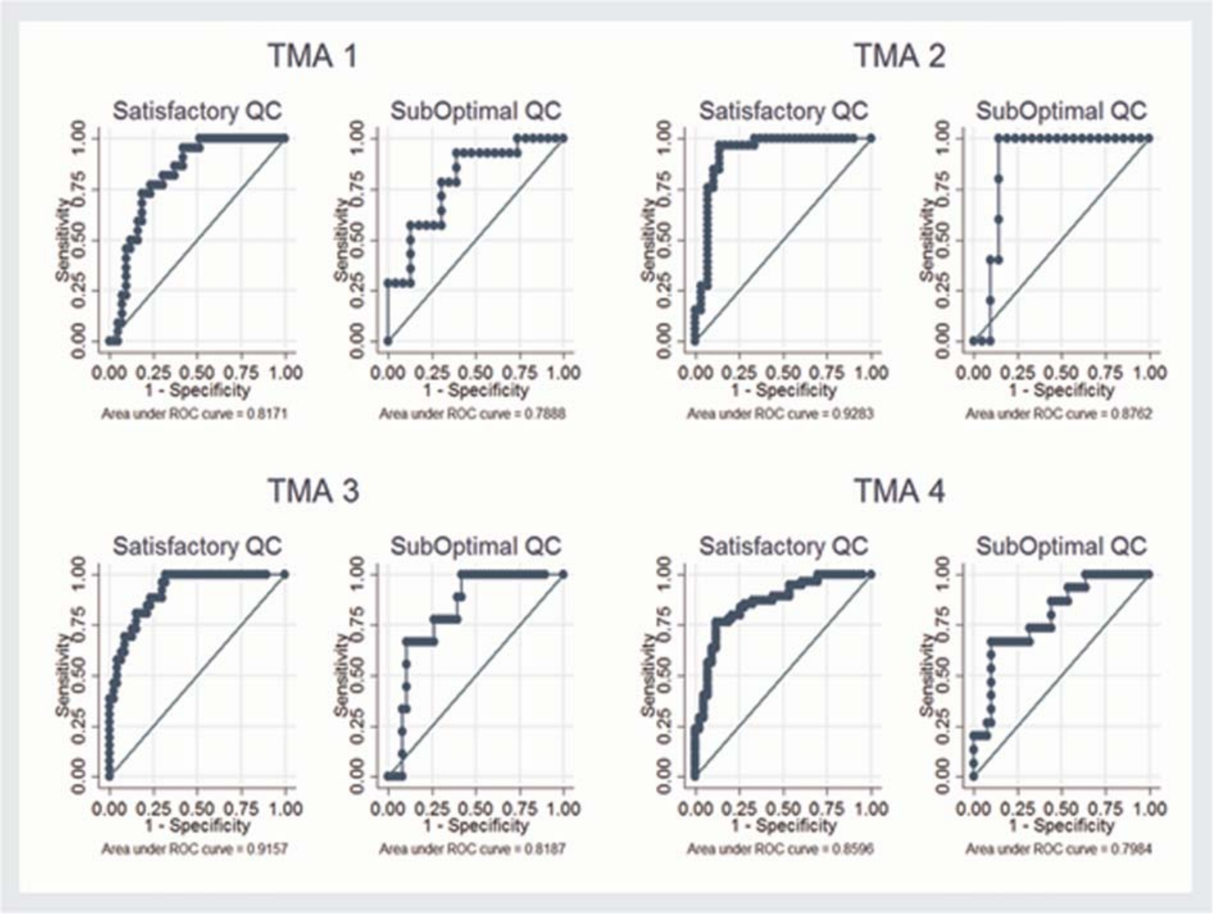

Supplementary Figure S2A (TMAs 1–15 & overall).: Graphs comparing the ROC curves for the discriminatory accuracy of the automated continuous scores against categories of the visual score by QC status among all 15 TMAs in the training set and overall.

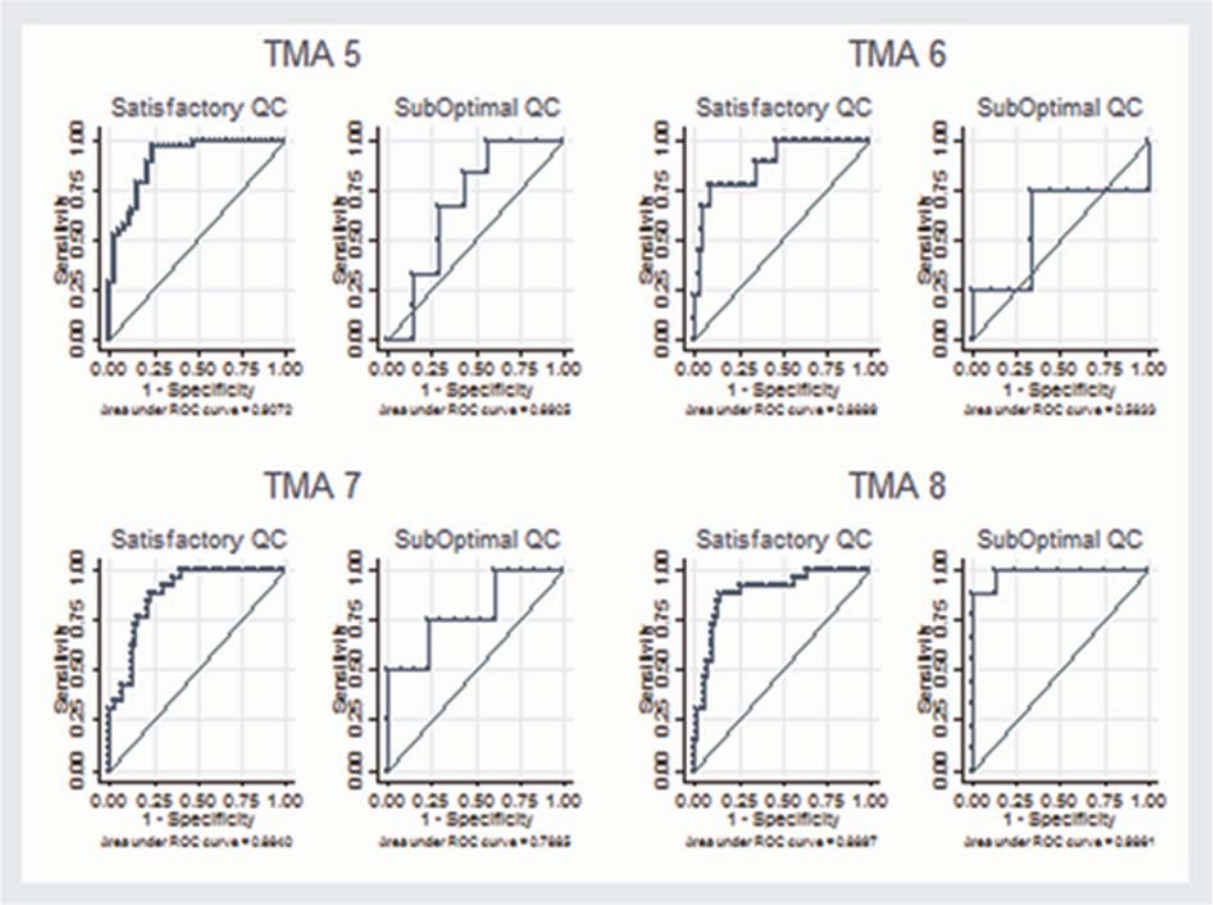

Supplementary Figure S2B (TMAs 1–15 & overall).: Graphs comparing the ROC curves for the discriminatory accuracy of the automated continuous scores against categories of the visual score by QC status among all 15 TMAs in the training set and overall.

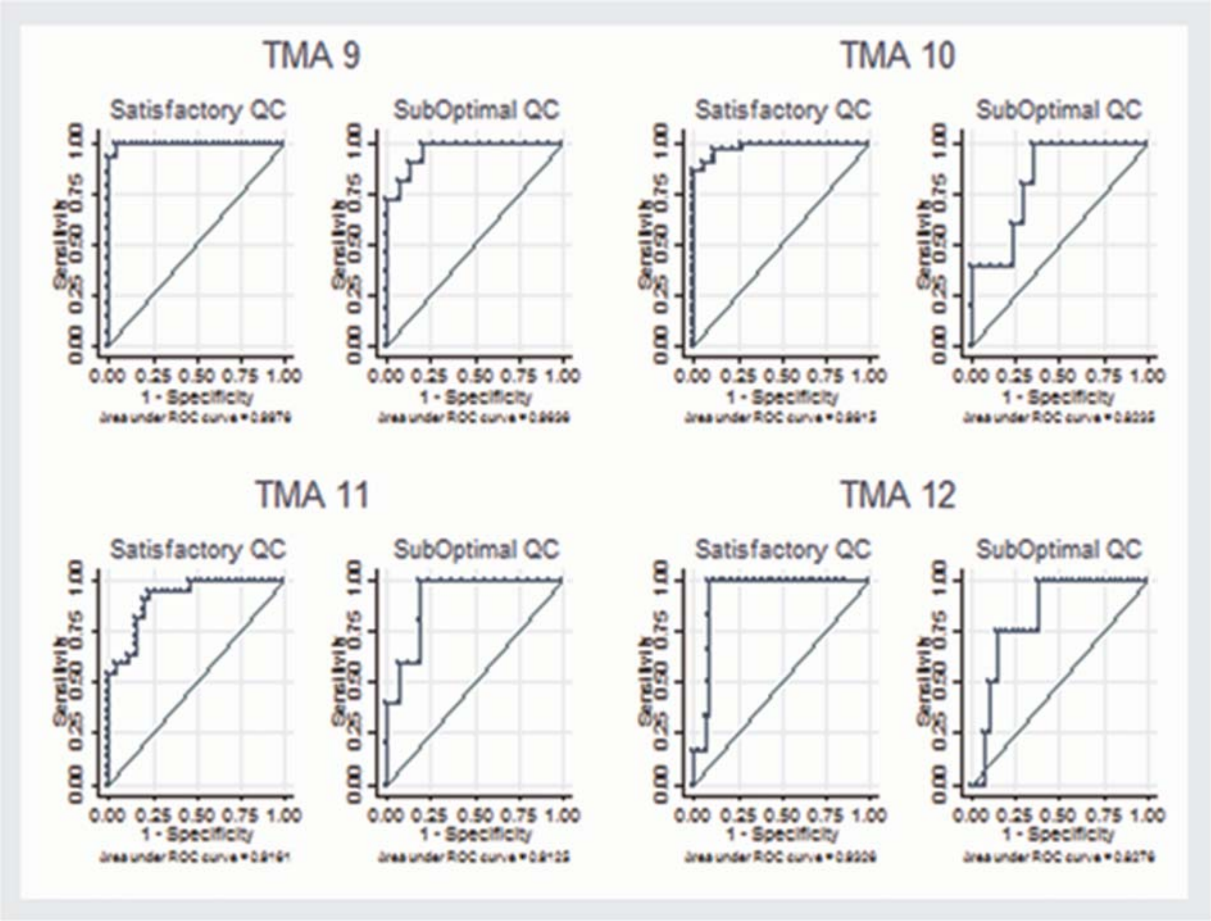

Supplementary Figure S2C (TMAs 1–15 & overall).: Graphs comparing the ROC curves for the discriminatory accuracy of the automated continuous scores against categories of the visual score by QC status among all 15 TMAs in the training set and overall.

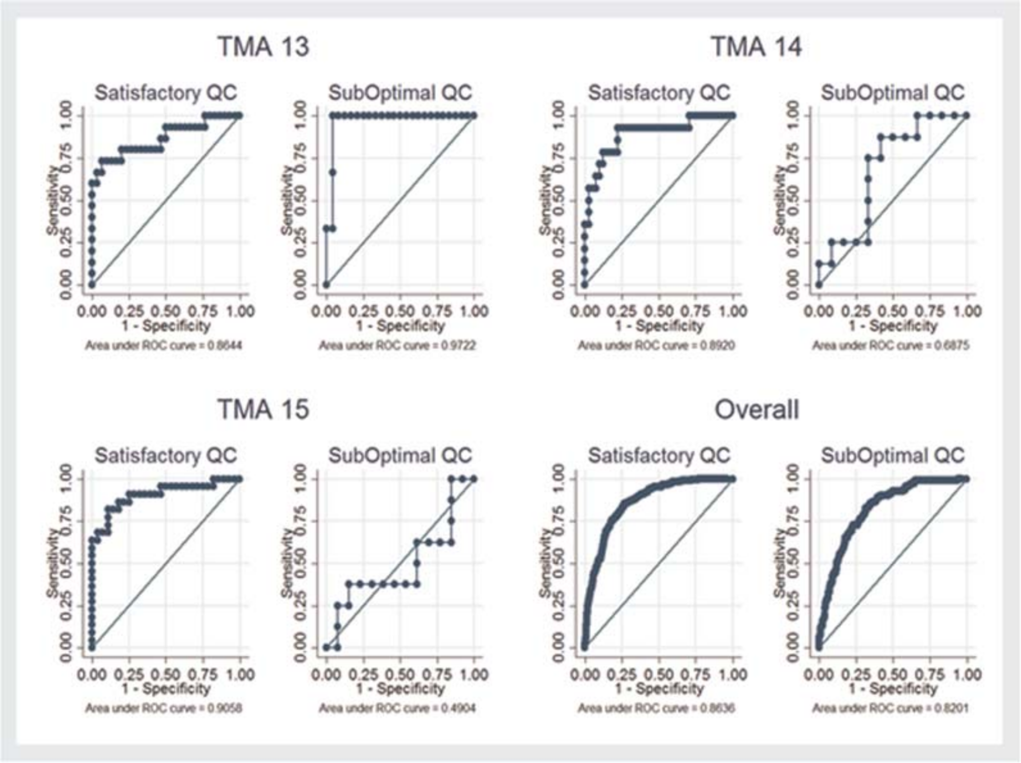

Supplementary Figure S2D (TMAs 1–15 & overall).: Graphs comparing the ROC curves for the discriminatory accuracy of the automated continuous scores against categories of the visual score by QC status among all 15 TMAs in the training set and overall.
