# Supplementary figures and images for: High‐throughput automated scoring of Ki67 in breast cancer tissue microarrays from the Breast Cancer Association Consortium
Source: J Pathol Clin Res. 2016 Apr 6;2(3):138–53. doi: 10.1002/cjp2.42 (PMC4958735; doi:10.1002/cjp2.42)

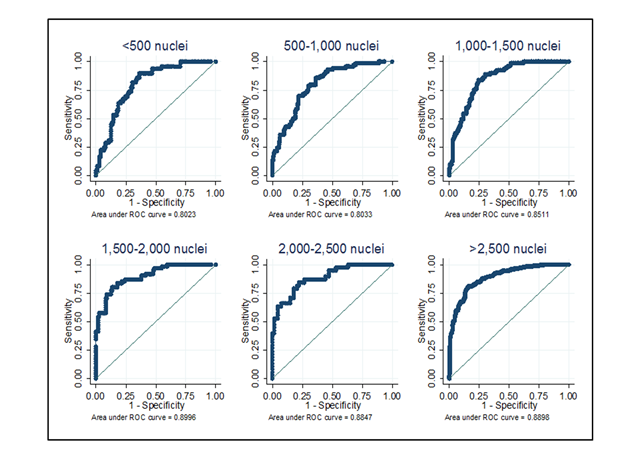

Supplement: Supplementary file 4 — Figure S3. ROC curves, by total nuclei count, for the discriminatory accuracy of the automated quantitative Ki67 scores against categories of the visual score [file CJP2-2-138-s003.tif]

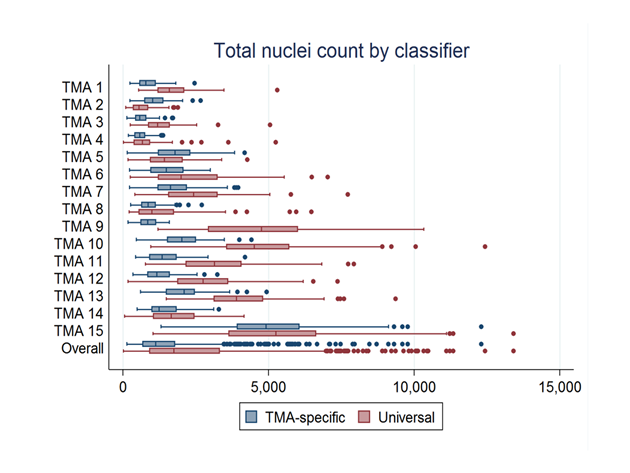

Supplement: Supplementary file 5 — Figure S4. Distribution of total nuclei counted by the machine for the TMA‐specific and universal classifiers among the 15 TMAs in the training set and overall [file CJP2-2-138-s004.tif]

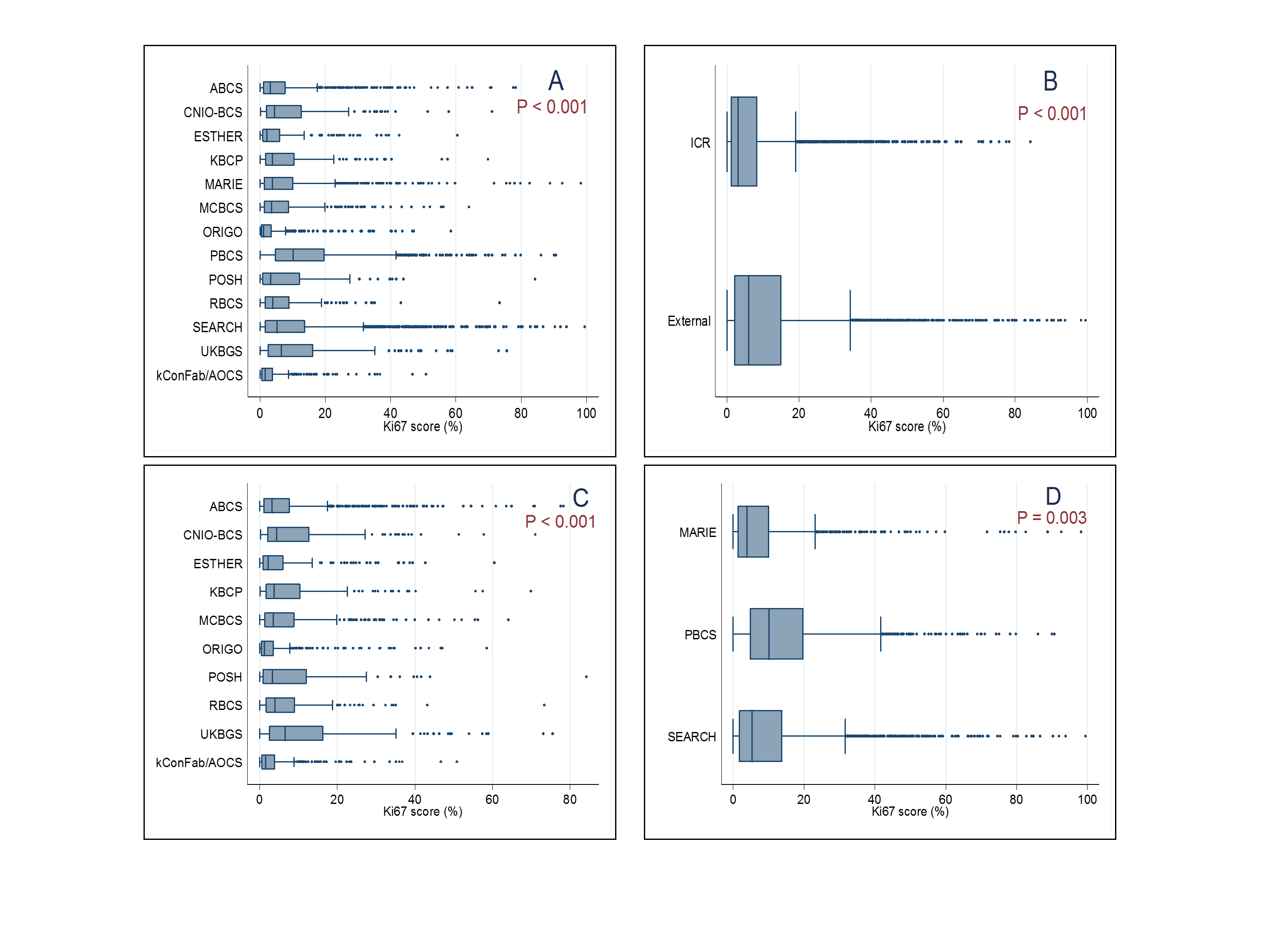

Supplement: Supplementary file 6 — Figure S5. Distribution of the subject level (N=9,059) Ki67 score among (A) the different study groups, (B) according to whether the TMAs were stained at the ICR or in an external location, (C) among study groups whose TMAs were stained at the ICR and (D) among study groups whose TMAs were stained in an external location [file CJP2-2-138-s005.tif]

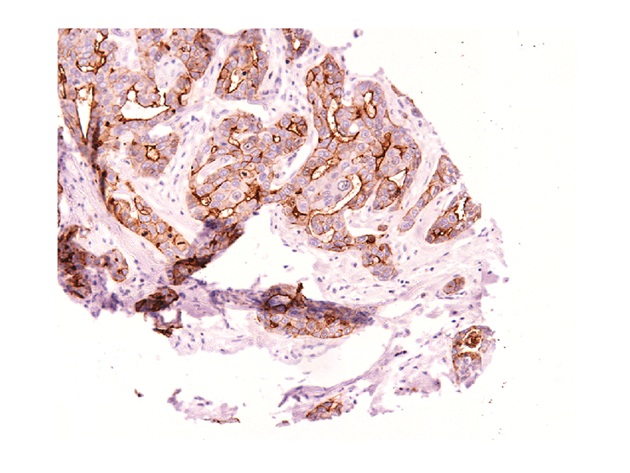

Supplement: Supplementary file 7 — Figure S6. Screengrab for a representative core in which discrepancy (ie, visual category 1 and Ariol category 4) between visual and automated scores was observed. The most common causes of ‘false positive’ by the machine are related to quality control: more specifically, the presence of background staining, core folding and membrane (instead of nuclear) staining. Of these, membrane staining was more prevalent and was observed in 8.7% of the cores [file CJP2-2-138-s006.tif]

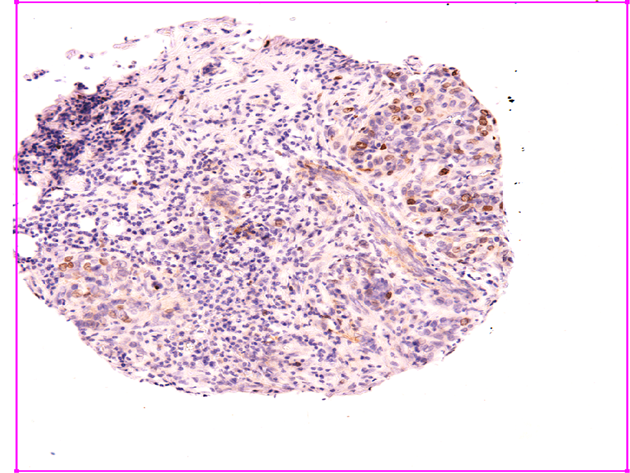

Supplement: Supplementary file 8 — Figure S7. Screengrab for a representative core in which discrepancy (ie, visual category 4 and Ariol category 1) between visual and automated scores was observed. The most common causes of ‘false negatives’ by the machine include marked lymphocytic infiltration with only occasional nests of invasive malignant cells, poor fixation, nuclear halo, and very low intensity DAB [file CJP2-2-138-s007.tif]
